# Population-based estimates of different dosage types of psychedelic use across socio-demographic groups in Germany

**DOI:** 10.1038/s41598-025-03873-0

**Published:** 2025-05-29

**Authors:** Sebastian Sattler, Suzanne Wood, Margit Anne Petersen, Fiona Seiffert, Guido Mehlkop

**Affiliations:** 1 Faculty of Sociology, Bielefeld University, 33615 Bielefeld, Germany; 2 Center for Uncertainty Studies (CeUS), Bielefeld University, 33615 Bielefeld, Germany; 3 Pragmatic Health Ethics Research Unit, Institut de recherches cliniques de Montréal (IRCM), Montréal, H2W 1R7 Canada; 4 Department of Psychology, University of Toronto, Toronto, M5S 3G3 Canada; 5 Center for Alcohol and Drug Research, Aarhus Universitet, Copenhagen, 2400 Copenhagen Denmark; 6 Faculty of Management, Economics and Social Sciences, University of Cologne, Cologne, 50923 Germany; 7 Faculty of Economics, Law and Social Sciences, University of Erfurt, Erfurt, 99089 Germany; 8 Institute for Planetary Health Behaviour, University of Erfurt, Erfurt, 99089 Germany

**Keywords:** Human behaviour, Risk factors

## Abstract

Psychedelic drugs, particularly taking small amounts of psychedelics in a cyclical pattern over days (so-called microdosing), have garnered growing scientific and public interest, but representative data on different dosage levels is scarce. To better understand this trend, we surveyed a nationwide sample of 11,299 adults in Germany. The survey assessed lifetime and past six-month psychedelic use by dosage as well as socio-demographic variables (sex, age, education, employment status, household equivalence income, partner arrangements, and place of residence). Results show that 5.0% of respondents self-reported lifetime psychedelic use, while 0.7% reported past six-month use. Medium to high dosing was more prevalent than microdosing. Moreover, high probabilities of using multiple forms of psychedelics were uncovered. We also observed variation in use across socio-demographic groups. For example, psychedelics use was less likely in females than males, and older than younger respondents. Past six-month microdosing was less likely in rural areas, and past six-month medium to high dosing was less prevalent in individuals with higher income or who live with a partner. This study shows limited support for widespread use in Germany and highlights diverse usage patterns across socio-demographics. These findings can inform policies, especially considering the overlap in usage of various substances.

**Supplementary Information:**

The online version contains supplementary material available at 10.1038/s41598-025-03873-0.

## Introduction

A resurgence of interest in psychedelic and related substances, often referred to as a “Psychedelic Renaissance”,^1^ has gained momentum in recent years. After a peak in popularity in the 1950s and 1960s, empirical research ground to a halt due to prohibition, lack of production of a standard supply, among other factors,^2^ leaving many fundamental aspects of psychedelics unclear. Today, the field still lacks consensus on questions ranging from basic mechanisms of action to population-based usage patterns. Therefore, it is imperative that researchers across the medical and social sciences work together to characterize the current resurgence at all levels of analysis, from both clinical and non-clinical perspectives.

Researchers in the medical sciences generally agree on the relatively untapped potential that psychedelic research has for future mental health treatment (e.g., to treat depression, addiction, or post-traumatic stress disorder), including scholars in psychedelic public health, voicing concerns about equitable access to these potentially useful health-promoting tools^3^. This scientific hope and excitement, however, may also generate increasing interest and usage among the general public before psychedelics are approved for clinical use^4–10.^ Regardless, this optimism has led governments to ease research restrictions, decriminalize and legalize psychedelics, and has led venture capital funds to invest in psychedelics research^11–14^. Forecasts of the growing psychedelic global drug market value predict a range from $6.82 billion USD^15^ to $7.2 billion USD^16^ in 2029.

Still, in most countries, use of psychedelics outside of clinical settings remains under strict prohibition, leading to additional challenges in conducting basic research, identifying users, and accurately estimating the prevalence of use of different substances and dosages. Some countries, such as the United States and Australia, are softening bans on select psychedelics, including psilocybin, while LSD restrictions have remained mostly untouched^11^. In Germany, the country in which the current study was conducted, psychedelic and related substances are mainly illegal and discussed for medical use only^17.^ After the (regulated) legalization of cannabis in 2024, further legalization of other illicit substances (including psychedelics) is not planned. While media in Germany seem to more frequently discuss therapeutic uses of psychedelic substances,^18,19^ also the non-medical use for recreational reasons or enhancement purposes receives attention^20,21.^ Therefore, it is worth investigating whether such attention finds traction in prevalence rates, supporting the expectation of psychedelics becoming a kind of “folk drug”^22^.

What is considered a psychedelic substance varies by research fields and includes both naturally occurring and chemically produced substances. Among them are more “classic” serotonin 2 A receptor (5-HT_2A_R) agonists (e.g., psilocybin, lysergic acid diethylamide (LSD), N,N-Dimethyltryptamine (DMT), and mescaline); mixed serotonin and dopamine reuptake inhibitors and releasers (i.e., empathogens or entactogens such as the stimulant, 3,4-Methylenedioxy methamphetamine (MDMA)); glutamate receptors antagonists (i.e., dissociative anaesthetic agents such as ketamine); and atypical psychedelics with various mechanisms of action (e.g., ibogaine or tetrahydrocannabinol (THC))^23–25^. Research examining different doses of psychedelic substances reveals a clear difference in subjective effects. While there is discussion and also disagreement about how doses could be defined,^[e.g., 26,27^ in this study, we divided them into two broad categories, although we acknowledge that more fine-grained categories exist^[e.g., 28^ (see “Limitations” section): microdoses and medium to high doses. Microdosing psychedelics involves consuming a small amount of the substance in a cyclical pattern over periods of days^29^. Some scholars define this amount to be about one-tenth to one-twentieth of recreational doses, depending on the substance^28^. The acute subjective effects experienced by users range from being subtle to imperceptible; the desired benefits of this form of psychedelic consumption tend to revolve around overall boosts in mood, concentration, creativity, among others^30–33^. Medium to high doses of psychedelics lead to noticeable and, at times, profound subjective effects. Users of these higher doses tend to seek the experience of alterations in mood and perception, including hallucinations, along with mystical or spiritual experiences^34^.

While generally considered safe substances, psychological (for example anxiety, paranoia, suicidality) as well as physical side effects (such as hypertension and cardiovascular effects or, even more rarely, death) have been reported^30,35,36^. Medical risks of classic psychedelics are often minimal; one study compared the need for medical help in relation to substances used and deemed psilocybin among the safest^37^. A meta-analysis found that side effects such as increased heart rate and blood pressure as well as feelings of nausea and anxiety were common during the acute phase of psychedelic administration, but subsided within hours^38.^. This analysis focused on clinical studies that employed ayahuasca, LSD and psilocybin. Research on use of these classic psychedelics outside clinical settings has found similar results. Substances such as MDMA and ketamine have very different risk profiles, and therefore the risks and adverse effects of these may not be considered as safe, even if they are sometimes used in similar ways as classic psychedelics. For example, the combination of esketamine nasal spray and antidepressants has been shown to lead to clinical benefits for those with treatment-resistant depression, compared to antidepressants, alone^39^. Side effects, such as feelings of dissociation and dizziness, were transient and typically subsided within approximately two hours of esketamine administration. Use of ketamine outside clinical settings is associated with multiple risks, such as paranoia, panic, psychosis, cardiorespiratory problems especially when used in combination with other drugs, and fatal overdoses^40^. Similar risks are described with MDMA, particularly in high doses, such as anxiety and panic attacks, seizures, arrhythmia and hyperthermia, sexual risk-taking, damaged brain functions or death (although some deaths may be due to drug interactions, hypothermia, or other, related factors, rather than an overdose)^41^.

Identifying current patterns of use is a good first step to better characterize this possible health concern. Much of the older research generally recruits self-identified users to uncover their usage patterns. Current prevalence data often uses self-selection sampling,^42^ leaving representative data scarce. Also, existing studies often do not distinguish between microdosing and medium to high dosing,^43^ which are very different usage patterns with different risk profiles. Yet, identifying human usage patterns is one critical but under-addressed avenue of research. Especially detailing the specific substances used and under what dosing regimens, for example, contrasting medium to high doses with microdosing, are essential questions when characterizing population-wide usage patterns. Because the category of psychedelics is contested and thus unclear, asking about different substances separately but in the same study may reveal new aspects. Moreover, demographic analyses have been conducted within populations who use,^29^ while general demographics are also needed.

Current research in Germany is beginning to paint a picture of the general characteristics of today’s users. A recent representative study found a 3.4% lifetime and 0.6% 12-month prevalence for LSD, a 4.5% lifetime and 0.5% 12-month prevalence for hallucinogenic mushrooms and a 4.0% lifetime and 1.3% 12-month prevalence for new psychoactive substances among German adults (18–64 years)^44^. However, this study was conducted during the COVID pandemic, a time frame that might not be representative of non-pandemic situations^45^. In 2018, the respective prevalences were 2.1% for lifetime and 0.3% for past 12-month use of LSD, 3.0% for lifetime and 0.4% for past 12-month use of hallucinogenic mushrooms and 2.6% for lifetime and 0.9% for past 12-month use of new psychoactive substances^46^. While this study provides important insights, it does not differentiate between medium to high doses and microdosing.

Therefore, the aim of this article is to contribute towards characterizing the current patterns of psychedelic use by adults in Germany, using population-based data, focusing on multiple substances that fall under the category of psychedelics as well as on different dosage-types.

## Methods

### Sample

This study used data from the nationwide web-based *ENHANCE*-study with adults (18 years and older) in Germany. Recruitment of the initial sample of this four-wave study took place offline and targeted a nationally representative group, regarding sex, age, education, and federal state for the adult residential population having Internet access (applying to 92% of all households)^47^. We used data of wave four (conducted between October and December, 2022) since only here questions related to psychedelics have been assessed. In this wave, 28,567 individuals were invited to participate in this voluntary and pseudonymized survey, in which the researchers had no access to the respondents’ personal data. We also invited individuals who declined their participation in waves two and/or three as well as a refreshment sample of 10,104 individuals to compensate for demographic imbalances due to selective participation of hard-to-reach participants. In total, 13,452 individuals consented to participate, while 12,168 completed the survey and received bonus points (worth approximately $3.00) as an incentive. These points could be exchanged for vouchers, charity lottery tickets, or donations to UNICEF. The analytic sample included 11,299 individuals who responded to all variables used (49.1% female_weighted_, mean age_weighted_: 48.93 years; see Table 1 for all descriptive statistics). This study was approved by the Ethics Committee of the University of Erfurt (reference number: EV-20220830). Our work aligns with the Code of Ethics of the American Sociological Association (ASA) and although our study is not a medical study, we adhere to the Code of Ethics of the World Medical Association (Declaration of Helsinki) to protect human research participants.

**Table 1:** Sample description without and with sampling weights (*N* = 11,299).

	Unweighted frequency	Unweighted proportion (in %)	Weighted proportion (in %)
**Sex**			
Male	5,773	51.1	50.9
Female	5,526	48.9	49.1
**Age**			
18–29	1,220	10.8	14.4
30–39	2,278	20.2	18.9
40–49	1,729	15.3	14.2
50–59	2,694	23.8	23.6
60+	3,378	29.9	28.9
**Education**			
Lower than university entrance qualification	4,695	41.6	59.2
At least university entrance qualification	6,604	58.4	40.8
**Employment status**			
Full-time	5,721	50.6	47.1
Part-time	2,401	21.2	21.7
In education	407	3.6	5.5
Not employed	2,770	24.5	25.7
**Income**			
Low ( < = 60% median)	1,735	15.4	19.7
Medium	9,037	80.0	77.1
High (> 2*median)	527	4.7	3.2
**Partner living in the household**			
No	3,785	33.5	36.9
Yes	7,514	66.5	63.1
**Place of residence**			
Urban	8,037	71.1	70.7
Rural	3,262	28.9	29.3

### Measures

*Self-reported use of psychedelics.* Respondents were asked to self-report their prior use of psychedelics, including LSD (also labeled “acid” in the questionnaire) or LSD analogues (e.g., 1P-LSD or ETH-LAD), psilocybin (“magic mushrooms”), as well as other psychedelics (e.g., DMT and ayahuasca) and substances that can be used in similar ways (e.g., ketamine and MDMA). We explained to the respondents that these drugs are taken in varying doses. With a medium to high dose, they can cause perceptual changes (e.g., seeing or hearing things in a new way) or hallucinations (e.g., seeing or hearing things that are not present in the environment). With microdosing—that is, a very small dose—some people report subtle changes in their perception over a long period of intermittent use, whereas others detect no noticeable changes. We asked if respondents had ever taken (1) LSD (“acid”) or LSD analogues (e.g. 1P-LSD or ETH-LAD), (2) Psilocybin (“magic mushrooms”), and (3) Other psychedelic drugs (e.g. DMT, ketamine, MDMA, ayahuasca) “in medium to high dose” or “in microdose”. Response options were “no, never”, “yes, more than 6 months ago”, “yes, in the last 6 months but not in the last 30 days”, and “yes, in the last 30 days”. Also “no response” was possible (chosen by 0.8-0.9% of respondents per item). Respondents indicating other psychedelic drug use received a follow-up open-ended question to indicate which drug(s) they had been using “in medium to high dose” or “in microdose”. Based on these questions, six dichotomous variables have been created indicating (a) lifetime and (b) past-six months use of any of these substances, irrespective of the dose, (c) lifetime and (d) past six-month microdosing, as well as (e) lifetime and (f) past six-month medium to high dosing (each time coded as “no” [0] and “yes” [1]).

*Sociodemographic characteristics.* We assessed the respondents’ sex, age, education, employment status,^[cf., 48^ and whether they had a partner living in their household (see Table 1). The estimated equivalence household net income was measured with an open-ended question. If no answer was provided, we showed income categories^[cf., 48^ and used the mean value of these categories, while for the last open-ended category (“20,000 and more”), the value 20,000 was used^49^. We computed the equivalence income by considering the number of household members with the OECD-modified scale^50^ for which the first adult received a weight of 1, each additional adult received a weight of 0.5, and each child (under 14 years) received a weight of 0.3. This weight was used as a divisor for the income. Moreover, place of residence was assessed. Therefore, we categorized individuals as living either in a rural or urban settlement structure^51^, based on their postal codes.

### Pretesting

We conducted qualitative cognitive pretests (*N* = 10) with a think-aloud approach and probing questions^52^ as well as a quantitative pretest (*N* = 205) to examine and improve the clarity and accuracy of all measures. The findings of these pretests suggest that the materials used were clear, understandable, and suitable for the nationwide sample.

### Statistical analysis

To examine overlaps of patterns of psychedelic use, we computed percentages and Pearson´s correlations (identical to *Phi* for bivariate measures) for which *r* > ± 0.2 indicates small effects, *r* > ± 0.5 medium effects, and *r* > ± 0.8 large effects^53^. We used logistic regression models^54^ to examine the differences in the self-reported prevalence across sociodemographic characteristics. For categorical variables with more than two groups (e.g., age), we rotated the reference group to enable a comparison of all groups (see Tables S1-6, Supplementary Material). We present unadjusted odds ratios (*ORs*) to indicate the magnitude of bivariate associations between the prevalence measures and each sociodemographic group as well as adjusted *ORs*, which control for all other variables. An *OR* ≥ 1.50 (≤ 0.67) indicates a small effect, an *OR* ≥ 2 (≤ 0.5) a medium effect, and an *OR* ≥ 3 (≤ 0.33) a large effect^53^. The *ORs* are presented along with their 95% confidence intervals. Across all analyses, we use sampling weights to increase the representativity of the sample (see Table 1). The weights are computed with iterative proportional fitting^55^ on the basis of sex, age, education, and federal state.

## Results

### Prevalence of use: substances and doses

**Fig. 1:**
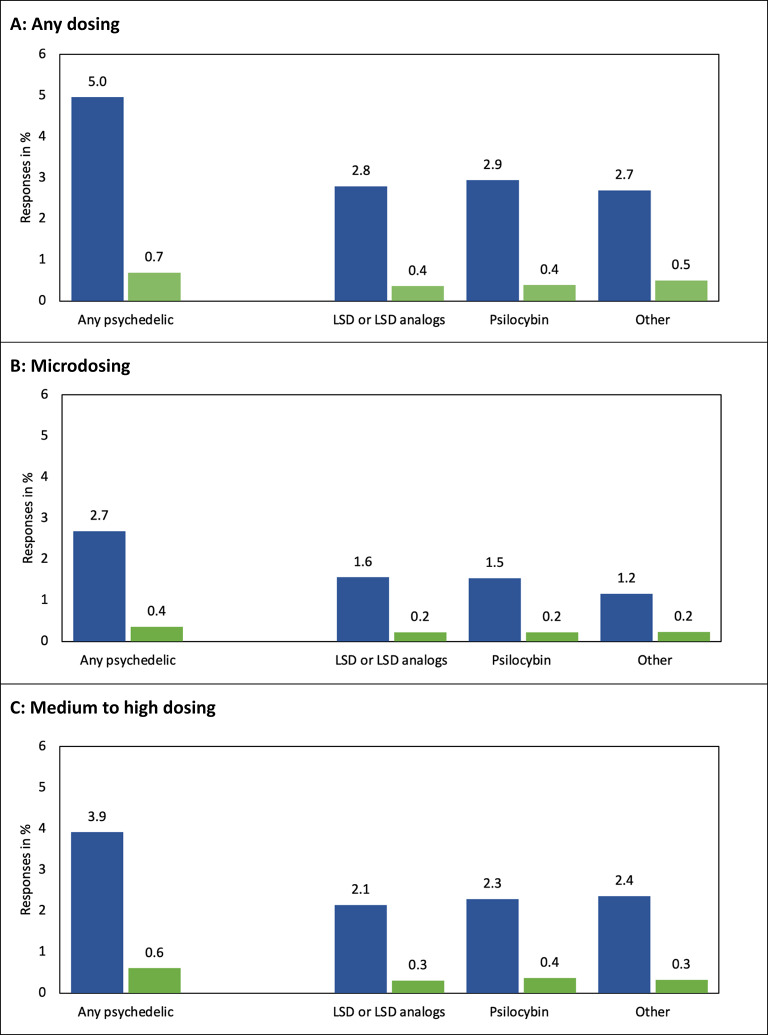
Lifetime (Blue bars) and past six-month (Green bars) prevalence of using psychedelic drugs in percent (*N* = 11,299)^a^ The category “any psychedelic” is based on the substance-specific data, indicating that the respondent reported the use of at least one of the three categories of psychedelics.

Panel A in Fig. 1 shows that 5.0% of respondents self-reported any psychedelic use during their lifetime, irrespective of dosing, while 0.7% did so for the past six months. More specifically, roughly 3% used LSD or LSD analogs, psilocybin, or other substances in their lifetime and half a percent during the past six months. Panels B and C show that use of medium to high doses seems more prevalent in Germany than microdosing across all categories.

To investigate the overlap in use of different psychedelics across dosing and time of use, the upper sections of Table 2 (lifetime) and Table 3 (past six-month) provide percentages of overlaps, and the lower sections provide correlation coefficients between the measures. The results suggest that 58.7% of lifetime users of LSD (any dose) also used psilocybin (any dose) during their lifetime, while 55.7% of lifetime users of psilocybin (any dose) also engaged in LSD (any dose) during their lifetime. Of all past six-month medium to high LSD users, 67.8% also engaged in medium to high dosing of psilocybin within this period, while only 55.2% of past six-month users of psilocybin (medium to high dosing) also used LSD (medium to high dosing) in the respective period.

**Table 2:** Overlap and correlations among different psychedelic use outcomes – lifetime use (*N* = 11,299).

	1)	2)	3)	4)	5)	6)	7)	8)	9)
**Overlap in percent (with 95% confidence intervals in brackets)** ^a^									
**1)** LSD *(any dosing)*		56.0 [48.9;62.9]	77.0 [70.8;82.2]	58.7 [51.6;65.4]	31.7 [24.7;39.7]	51.3 [44.1;58.4]	54.2 [47.1;61.2]	26.2 [19.7;34.0]	49.7 [42.5;56.9]
**2)** LSD *(microdosing)*	100		58.9 [49.0;68.1]	62.9 [53.5;71.4]	52.8 [42.8;62.5]	52.5 [42.5;62.3]	55.6 [45.7;65.1]	42.4 [32.3;53.1]	48.4 [38.3;58.6]
**3)** LSD *(medium to high dosing)*	100	42.9 [34.6;51.6]		63.3 [55.2;70.7]	30.2 [22.1;39.6]	61.3 [53.1;68.9]	58.6 [50.4;66.4]	25.1 [17.7;34.4]	57.5 [49.3;65.4]
**4)** Psilocybin *(any dosing)*	55.7 [48.8;62.3]	33.4 [26.7;41.0]	46.3 [39.3;53.3]		52.6 [45.7;59.4]	77.9 [72.0;82.8]	51.9 [45.0;58.7]	24.3 [18.1;31.9]	48.4 [41.4;55.4]
**5)** Psilocybin *(microdosing)*	57.3 [47.4;66.7]	53.3 [43.2;63.2]	41.9 [31.6;52.9]	100		57.9 [47.9;67.4]	49.7 [39.4;59.9]	41.0 [30.7;52.1]	44.6 [34.3;55.4]
**6)** Psilocybin *(medium to high dosing)*	62.5 [54.7;69.7]	35.8 [27.9;44.6]	57.5 [49.6;65.1]	100	39.1 [31.2;47.8]		61.0 [53.4;68.1]	27.5 [20.0;36.5]	59.5 [51.8;66.7]
**7)** Other *(any dosing)*	56.2 [48.8;63.4]	32.3 [25.2;40.3]	46.8 [39.4;54.4]	56.7 [49.3;63.7]	28.5 [21.5;36.9]	51.9 [44.4;59.3]		43.3 [36.0;50.9]	87.8 [83.2;91.3]
**8)** Other *(microdosing)*	62.8 [52.0;72.4]	56.9 [45.6;67.5]	46.4 [34.7;58.5]	61.5 [50.7;71.2]	54.5 [43.1;65.4]	54.0 [42.6;65.0]	100		71.9 [61.9;80.1]
**9)** Other *(medium to high dosing)*	58.7 [50.5;66.4]	32.0 [24.3;40.8]	52.3 [44.1;60.4]	60.1 [52.2;67.6]	29.2 [21.5;38.3]	57.6 [49.6;65.3]	100	35.4 [27.7;44.0]	
**Pairwise correlations** ^**b**^									
**1)** LSD *(any dosing)*									
**2)** LSD *(microdosing)*	***0.74***								
**3)** LSD *(medium to high dosing)*	**0.87**	*0.49*							
**4)** Psilocybin *(any dosing)*	***0.56***	*0.45*	***0.53***						
**5)** Psilocybin *(microdosing)*	*0.41*	***0.52***	*0.34*	***0.72***					
**6)** Psilocybin *(medium to high dosing)*	***0.55***	*0.42*	***0.58***	**0.88**	*0.47*				
**7)** Other *(any dosing)*	***0.54***	*0.41*	***0.51***	***0.53***	*0.36*	***0.55***			
**8)** Other *(microdosing)*	*0.40*	*0.48*	*0.33*	*0.38*	*0.47*	*0.38*	***0.65***		
**9)** Other *(medium to high dosing)*	***0.53***	*0.38*	***0.54***	***0.53***	*0.35*	***0.58***	**0.94**	***0.50***	

^a^Numbers represent the proportion of people to whom both the row information and column information apply (e.g., the number in the second row and the fifth column indicates that 52.8% of those who reported microdosing with LSD also reported microdosing of Psilocybin). ^b^Pearson’s coefficients are presented. Small effects (*r* > ± 0.2) are displayed in *italics*, medium effects (*r* > ± 0.5) in ***bolded italics***, and large effects (*r* > ± 0.8) in **bold**.

**Table 3:** Overlap and correlations among different psychedelic use outcomes – past six-month use (*N* = 11,299).

	1)	2)	3)	4)^c^	5)	6)^c^	7)	8)	9)
**Overlap in percent (with 95% confidence intervals in brackets)** ^a^									
**1)** LSD *(any dosing)*		62.2 [36.3;82.6]	84.9 [65.9;94.2]	64.0 [40.1;82.6]	43.2 [18.2;72.2]	64.0 [40.1;82.6]	70.2 [47.4;86.0]	37.1 [13.5;68.9]	41.6 [19.3;68.0]
**2)** LSD *(microdosing)*	100		75.7 [45.2;92.2]	73.5 [43.6;90.9]	65.5 [33.2;87.9]	73.5 [43.6;90.9]	76.4 [47.4;92.1]	55.7 [21.5;85.2]	30.5 [8.9;66.4]
**3)** LSD *(medium to high dosing)*	100	55.4 [26.5;81.1]		67.8 [41.5;86.2]	45.9 [17.9;76.7]	67.8 [41.5;86.2]	75.1 [50.3;89.9]	36.1 [10.7;72.8]	46.5 [19.7;75.5]
**4)** Psilocybin *(any dosing)*	58.6 [33.5;79.9]	41.8 [17.8;70.4]	52.7 [27.7;76.5]		56.9 [32.0;78.8]	95.5 [81.5;99.0]	74.7 [54.5;87.9]	42.1 [17.9;70.8]	40.5 [19.1;66.1]
**5)** Psilocybin *(microdosing)*	69.5 [30.1;92.3]	65.5 [27.2;90.6]	62.6 [24.8;89.5]	100		92.1 [67.3;98.5]	85.7 [60.1;96.0]	70.5 [32.8;92.1]	25.5 [6.1;64.1]
**6)** Psilocybin *(medium to high dosing)*	61.4 [35.4;82.2]	43.8 [18.8;72.4]	55.2 [29.2;78.6]	100	54.9 [29.1;78.4]		78.3 [58.7;90.1]	44.1 [18.9;72.7]	42.4 [19.7;68.8]
**7)** Other *(any dosing)*	49.9 [29.1;70.7]	33.8 [14.5;60.7]	45.4 [24.6;67.8]	58.1 [38.2;75.6]	37.9 [17.4;63.9]	58.1 [38.2;75.6]		46.1 [25.5;68.2]	64.5 [38.1;84.3]
**8)** Other *(microdosing)*	57.2 [22.9;85.8]	53.4 [19.8;84.2]	47.3 [15.0;82.1]	70.9 [40.8;89.7]	67.6 [36.4;88.4]	70.9 [40.8;89.7]	100		23.2 [7.9;51.6]
**9)** Other *(medium to high dosing)*	45.9 [26.7;66.4]	20.9 [7.7;45.7]	43.5 [24.4;64.8]	48.7 [29.4;68.4]	17.5 [5.3;44.3]	48.7 [29.4;68.4]	100	16.6 [7.5;32.7]	
**Pairwise correlations** ^**b**^									
**1)** LSD *(any dosing)*									
**2)** LSD *(microdosing)*	***0.79***								
**3)** LSD *(medium to high dosing)*	**0.92**	***0.65***							
**4)** Psilocybin *(any dosing)*	***0.61***	***0.55***	***0.60***						
**5)** Psilocybin *(microdosing)*	***0.55***	***0.65***	***0.53***	***0.75***					
**6)** Psilocybin *(medium to high dosing)*	***0.63***	***0.57***	***0.61***	**0.98**	***0.71***				
**7)** Other *(any dosing)*	***0.59***	***0.51***	***0.58***	***0.66***	***0.57***	***0.67***			
**8)** Other *(microdosing)*	*0.46*	***0.54***	*0.41*	***0.55***	***0.69***	***0.56***	***0.68***		
**9)** Other *(medium to high dosing)*	*0.44*	*0.25*	*0.45*	*0.44*	*0.21*	*0.45*	**0.80**	0.19	

^a^Numbers represent the proportion of people to whom both the row information and column information apply (e.g., the number in the second row and the fifth column indicates that 65.5% of those who reported microdosing with LSD also reported microdosing of Psilocybin). ^b^Pearson’s coefficients are presented. ^c^The broad similarity of columns 4) and 6) is due to the fact that the respective variables (Psilocybin, any dosing and Psilocybin, medium to high dosing) are very similar and that the only two respondents who make the difference do not use any other psychedelic in any dosing. Small effects (*r* > ± 0.2) are displayed in *italics*, medium effects (*r* > ± 0.5) in ***bolded italics***, and large effects (*r* > ± 0.8) in **bold**.

The correlations imply medium-sized positive associations between use of any dose of LSD, psilocybin, and other classic psychedelic substances (for both lifetime and past six-month use). For microdosing and medium to high dosing (lifetime and past six-month use), these associations are of small to medium size. The results further suggest associations of small to medium size between the uses of two doses, i.e., microdose and medium to high dose, for LSD, psilocybin, and other psychedelic substances across the lifetime. For the past six-month use, two associations of medium size were also uncovered, namely, for LSD microdosing and medium to high dosing as well as for psilocybin microdosing and medium to high dosing.

### Prevalence of use: demographic groups

#### Gender

Females were less likely to report the use of psychedelics across all indicators (see Tables 4 and 5). Bivariate effects were small in size for lifetime use of any dose, as well as for lifetime and past six-month microdosing, medium for past six-month use irrespective of dose, and for lifetime medium to high dosing, as well as close to large for past six-month medium to high dosing. After adjustment, most effects minimally increased.

#### Age

The bivariate analyses suggested higher prevalence rates among the two youngest age groups across all indicators and decreasing use with age (see also Tables S1-S6, Supplementary Material, for further comparisons of the other age categories not highlighted in the main text). The older age groups showed markedly lower prevalence rates; effect sizes were oftentimes large for comparisons against younger cohorts. After adjustment, these patterns remained relatively stable. There were exceptions, however; for example, the age group 30–39 years as compared to age group 18–29 years showed a lower past six-month microdosing with a medium effect size before adjustment, which vanished after adjustment. Moreover, several effects decreased in size, notably for the past six-month indicators.

**Table 4:** Unadjusted odds ratios (*UOR*) and adjusted odds ratios (*AOR*, each with 95% confidence intervals in brackets) for any dosing over the lifetime and the past six months^a^ (*N* = 11,299).

	Lifetime use UOR	Lifetime use AOR	Past six months UOR	Past six months AOR
**Sex**				
Male (ref.)				
Female	*0.60* [0.49;0.74]	*0.58* [0.45;0.73]	***0.41*** [0.20;0.82]	***0.41*** [0.20;0.84]
**Age**				
18–29 (ref.)				
30–39	1.16 [0.84;1.59]	1.18 [0.82;1.71]	*0.52* [0.25;1.10]	0.84 [0.37;1.92]
40–49	0.78 [0.54;1.13]	0.82 [0.52;1.28]	**0.18** [0.08;0.42]	**0.29** [0.10;0.84]
50–59	***0.43*** [0.30;0.63]	***0.45*** [0.29;0.70]	**0.05** [0.02;0.14]	**0.08** [0.02;0.27]
60+	**0.19** [0.12;0.29]	**0.17** [0.10;0.31]	**0.02** [0.00;0.07]	**0.02** [0.00;0.14]
**Education**				
Lower than university entrance qualification (ref.)				
At least university entrance qualification	*1.70* [1.37;2.12]	1.08 [0.85;1.38]	***2.52*** [1.23;5.16]	0.92 [0.41;2.05]
**Employment status**				
Full-time (ref.)				
Part-time	0.81 [0.61;1.07]	1.17 [0.87;1.58]	*1.60* [0.65;3.89]	*1.95* [0.79;4.79]
In education	1.11 [0.74;1.68]	0.85 [0.52;1.39]	***2.61*** [1.22;5.57]	0.80 [0.33;1.96]
Not employed	***0.44*** [0.31;0.63]	1.22 [0.78;1.92]	**0.28** [0.10;0.77]	1.04 [0.34;3.17]
**Equivalence income**				
Low (< 60% median) (ref.)				
Medium	1.01 [0.76;1.35]	1.06 [0.79;1.43]	***0.50*** [0.25;0.99]	0.78 [0.35;1.76]
High (> 2*median)	1.30 [0.80;2.11]	1.14 [0.69;1.88]	***0.42*** [0.11;1.54]	*0.56* [0.15;2.10]
**Partner in household**				
No partner (ref.)				
Partner	0.77 [0.62;0.96]	0.92 [0.71;1.18]	**0.21** [0.11;0.38]	***0.38*** [0.19;0.73]
**Place of residence**				
Urban (ref.)				
Rural	0.77 [0.60;0.99]	0.81 [0.63;1.04]	*0.64* [0.28;1.50]	0.76 [0.34;1.70]

^a^*UORs* refer to bivariate comparisons between groups, while *AORs* control for the other independent variables displayed in the table. Small effects (*OR* ≥ 1.50 and ≤ 0.67) are displayed in *italics*, medium effects (*OR* ≥ 2 and ≤ 0.5) in ***bolded italics***, and large effects (*OR* ≥ 3 and ≤ 0.33) in **bold**.

**Table 5:** Unadjusted odds ratios (*UOR*) and adjusted odds ratios (*AOR*, each with 95% confidence intervals in brackets) for microdosing and medium to high dosing over the lifetime and the past six months^a^ (*N* = 11,299^b^).

	Microdosing				Medium to high dosing			
	Lifetime *UOR*	Lifetime *AOR*	Past six months *UOR*	Past six months *AOR*	Lifetime *UOR*	Lifetime *AOR*	Past six months *UOR*	Past six months *AOR*
**Sex**								
Male (ref.)								
Female	*0.67* [0.50;0.89]	*0.63* [0.45;0.87]	*0.60* [0.21;1.75]	*0.58* [0.19;1.78]	***0.50*** [0.39;0.65]	***0.47*** [0.36;0.62]	***0.34*** [0.15;0.76]	**0.33** [0.14;0.77]
**Age**								
18–29 (ref.)								
30–39	1.43 [0.88;2.33]	*1.51* [0.87;2.59]	***0.50*** [0.13;1.87]	1.02 [0.29;3.68]	1.15 [0.81;1.64]	1.17 [0.78;1.74]	***0.48*** [0.22;1.04]	0.77 [0.32;1.84]
40–49	0.89 [0.54;1.48]	0.94 [0.52;1.71]	**0.27** [0.07;1.01]	*0.55* [0.13;2.40]	0.78 [0.52;1.17]	0.80 [0.48;1.31]	**0.11** [0.04;0.29]	**0.18** [0.05;0.58]
50–59	*0.57* [0.34;0.97]	*0.60* [0.33;1.09]	**0.09** [0.02;0.36]	**0.17** [0.03;0.91]	**0.33** [0.22;0.52]	***0.34*** [0.20;0.56]	**0.03** [0.01;0.10]	**0.05** [0.01;0.20]
60+	**0.19** [0.11;0.36]	**0.18** [0.08;0.40]	**0.03** [0.01;0.19]	**0.07** [0.01;0.48]	**0.15** [0.09;0.26]	**0.13** [0.07;0.26]		
**Education**								
Lower than university entrance qualification (ref.)								
At least university entrance qualification	*1.57* [1.17;2.09]	1.06 [0.77;1.44]	***2.23*** [0.73;6.84]	0.86 [0.26;2.89]	*1.76* [1.37;2.27]	1.07 [0.81;1.41]	***2.69*** [1.20;6.06]	0.88 [0.36;2.13]
**Employment status**								
Full-time (ref.)								
Part-time	0.83 [0.55;1.25]	1.18 [0.76;1.83]	***2.63*** [0.74;9.31]	**3.47** [1.00;12.00]	0.82 [0.59;1.14]	1.28 [0.91;1.80]	*1.77* [0.68;4.64]	***2.20*** [0.84;5.74]
In education	1.20 [0.70;2.05]	1.13 [0.57;2.25]	***2.64*** [0.85;8.18]	0.97 [0.24;3.89]	0.97 [0.62;1.51]	0.68 [0.40;1.14]	***2.90*** [1.29;6.54]	0.84 [0.33;2.13]
Not employed	***0.46*** [0.30;0.70]	1.25 [0.73;2.15]	**0.24** [0.07;0.91]	0.81 [0.17;3.90]	***0.44*** [0.29;0.66]	1.38 [0.82;2.30]	**0.26** [0.08;0.88]	1.18 [0.33;4.19]
**Equivalence income**								
Low (< 60% median) (ref.)								
Medium	0.94 [0.65;1.34]	0.95 [0.64;1.41]	*0.61* [0.19;1.94]	1.11 [0.29;4.29]	0.96 [0.69;1.33]	1.02 [0.73;1.43]	***0.48*** [0.23;1.02]	0.80 [0.33;1.95]
High (> 2*median)	0.92 [0.49;1.73]	0.79 [0.41;1.52]	0.68 [0.12;3.84]	1.18 [0.22;6.21]	1.39 [0.82;2.36]	1.26 [0.73;2.18]	***0.35*** [0.08;1.67]	*0.52* [0.11;2.47]
**Partner in household**								
No partner (ref.)								
Partner	0.91 [0.67;1.24]	1.07 [0.78;1.46]	**0.15** [0.06;0.37]	**0.21** [0.08;0.51]	0.70 [0.54;0.90]	0.85 [0.64;1.13]	**0.19** [0.10;0.37]	***0.40*** [0.19;0.86]
**Place of residence**								
Urban (ref.)								
Rural	0.91 [0.66;1.26]	0.94 [0.68;1.29]	*0.51* [0.13;2.00]	*0.61* [0.17;2.22]	0.81 [0.61;1.07]	0.86 [0.64;1.14]	0.76 [0.32;1.82]	0.91 [0.40;2.10]

^a^*UORs* refer to bivariate comparisons between groups, while *AORs* control for the other independent variables displayed in the table. ^b^For the past six-month analysis concerning medium to high dosing, the *UORs* for the different age categories and the *AORs* are based on 7,921 observations because among respondents aged 60 or older, no one indicated the respective psychedelic drug use. Small effects (*OR* ≥ 1.50 and ≤ 0.67) are displayed in *italics*, medium effects (*OR* ≥ 2 and ≤ 0.5) in ***bolded italics***, and large effects (*OR* ≥ 3 and ≤ 0.33) in **bold**.

#### Education

In the bivariate analyses, respondents with higher levels of education were more likely to report the use of psychedelics across all use indicators. While the effect sizes for all lifetime indicators were small, they were medium for all past six-month indicators. However, none of the effects remained substantial in the adjusted models.

#### Employment status

In the bivariate models, the lifetime and past six-month prevalence of use across all measures was lowest among those not employed compared to all other groups (see also Tables S1-S6, Supplementary Material). The effect sizes were mainly medium to large. Compared to full-time employment, the past six-month prevalence was higher among part-time employees and respondents in education with small to medium effect sizes. In the adjusted models, several bivariate effects decreased in size and partially turned to non-substantial effect sizes. Remaining substantial differences were, for example, the higher likelihood of reporting a six-month prevalence of any dosing, microdosing, and medium to high dosing for part-time employment compared to full-time, in education, and not employed, with small to large effects. In all analyses for lifetime use, effects turned non-substantial; only respondents in education were less likely to report medium to high dosing as compared to part-time and not employed respondents.

#### Equivalence income

Bivariate differences were non-substantial for all lifetime indicators across all three income groups (see also Tables S1-S6, Supplementary Material). However, for all past six-month use indicators, individuals in the low-income group were more likely to report any psychedelic use than those in the medium- and high-income group. The effects were mostly medium (with the exception of the comparison to the high-income group for microdosing, which slightly fell below the level of small effects). The medium and high-income group seemed to not substantially differ across all indicators. After adjustment, many effects for differences with the low-income group decreased and became non-substantial. However, few effects were still small in size, namely, the comparisons between the low- and high-income group for any dosing and for medium to high dosing in the past six months. Moreover, the comparison between high- and medium-income groups for medium to high dose, past six-month use showed a small effect size, with use less likely to be reported in the high-income group.

#### Partner

While there were no substantial bivariate differences in any of the lifetime indicators between people with and without partners in their households, those reporting to have such a partner were substantially less likely to report use across all indicators for past six-month use. These bivariate effects were large, but partially decreased to medium in adjusted models.

#### Place of residence

Generally, respondents from rural areas were less likely to report the use of psychedelics across all indicators. However, the bivariate differences were non-substantial, with the exception of past six-month use of any dose and microdosing. After adjustment, these effects slightly decreased.

### Sensitivity analysis

As a sensitivity analysis, we repeated the multivariate regression models by controlling for anonymity perceptions of the survey^56^ given the potentially sensitive nature of the topic. While most results remained stable (see Tables S7-S10, Supplementary Material), it is worth noting that for any dosing (past six-month), the part-time effect changed from small to medium and the high income effect changed from small to non-substantial; for microdosing (lifetime), the effect of the age group 30–39 changed from small to non-substantial; for microdosing (past six-month), the effect of the age group 40–49 (compared to 50–59) changed from large to medium, the high income effect turned to a small effect from a non-substantial effect; and for medium to high dosing (past six-month), the high income effects changed from small to non-substantial (compared to both low and medium income).

## Discussion

Our population-level data point to generally low usage rates of psychedelics in Germany. The rates of lifetime use of LSD (2.8%) and psilocybin (2.9%) in our sample are substantially lower than the rates reported by German respondents in the 2020 Global Drug Survey,^42^ wherein, 15.7% reported using LSD, and 18.1% reported using psilocybin within the past 12 months. It should be noted, however, that this Global Drug Survey was self-selective in that the target population consists of active drug users invited via media partners (e.g., newspapers and social media), making the data non-representative of any country’s general population^57^. Consequently, higher prevalence rates of psychedelics are expected among this sample in contrast to a probability-sample of the population. Compared to other studies in Germany using a representative population sample, our six-month prevalence of 0.4% for both LSD and psilocybin is very similar to the 12-month prevalence of 0.6% (2018: 0.3%) for LSD and 0.5% (2018: 0.4%) for hallucinogenic mushrooms found in 2021^46,58^. With regard to lifetime use of LSD, our prevalence of 2.8% is higher than the 2.1% reported by Seitz et al.^46^ but lower than the 3.4% found by Rauschert et al.^44^. For psilocybin, our sample showed a lifetime prevalence of 2.9%, which aligns closely with Seitz et al.’s^46^ finding of 3.0% but is notably lower than Rauschert et al.’s^44^ reported 4.5%. Overall, the pronounced discrepancy in usage rates between user-samples and representative or probability samples highlights the influence of recruitment methods and their unique role in population-based data collection.

Our data suggest that there are fewer users reporting self-administration of microdoses compared to medium to high doses. While the reason for this difference in prevalence should be further analyzed in future research, it might be that the concept of microdosing is a relatively new phenomenon, whereas taking higher doses is historically more common. The increased interest in microdosing in recent years might be a result of new societal challenges, such as digitalization, flexibilization of the labor market, and the increased prevalence of working environments like the home office that may combine work with childcare;^59,60^ these challenges might increase the demand for using microdoses of psychedelics as cognitive enhancers and to cope with stressors^61,62^. This would parallel self-reported reasons for use, which commonly revolve around expansion of awareness, coping, as well as enhancement^63^. Also, one international study found the most common reason for microdosing was performance enhancement,^30^ although it is worth noting that a recent double-blind, placebo-controlled study found no evidence for enhancement in participants administered microdoses over the course of two sessions^64^. Furthermore, microdosing psychedelics may be viewed as a phenomenon distinct enough from medium to high dosing to warrant a change in the way society sees psychedelics. Perhaps the lack of a significant perceptual change at the time of consumption will shift public opinion away from focusing on the use of psychedelics to merely get high and associated moral condemnation and stigma towards more positively viewed use for therapeutic or enhancement purposes.

The medium-sized prevalence of overlap in use of various psychedelics point to users being open to multiple substances and doses, rather than selectively using a single substance at a single dose. This finding is in line with a previous report of increased odds of medium to high dose psychedelic use in microdosers compared to non-microdosers^29^. Another study found that in their sample of users who had previously used a medium to high dose, almost 80% also reported microdosing^30^. These findings do not support the idea of a new generation of psychedelic users focused exclusively on microdosing for enhancement. In fact, a recent Danish study found that users of medium to large doses sometimes used microdoses in between the larger doses as a supplementary form of treatment and did not necessarily stay with the same substance^33^. A study using data from the Global Psychedelic Survey also concluded that co-using substances (e.g., alcohol, cannabis, stimulants, etc.) along with psychedelics is quite common, with other psychedelics being used in about half of the co-using participants^65^. Similarly, our data indicate that over half of LSD users of any dose also reported use of psilocybin, while over half of psilocybin users also reported using LSD. This finding should be considered when regulations of substances are being discussed, with current trends of decriminalization or legalization focusing slightly more on psilocybin than LSD^11^. These two substances could be used for similar purposes, although future research should investigate the reasons behind the use of specific psychedelics at specific doses.

Our findings also point to usage of psychedelics as a phenomenon that may be more prevalent in some population groups or communities (see Table S11, Supplementary Material, for a visual summary). The most robust demographic differences in the multivariate models relate to age and gender, such that, for most analyses, females and older respondents are less likely to have used psychedelics compared to males and 18- to 29-year-olds. Such patterns have often been observed in other studies for different substances and in different countries^44,46,66–70^. However, some of these studies rely on bivariate analysis (this restriction, along with the fact that effects are not always consistent across all investigated substances, also applies to several comparisons of use patterns for other demographics reported below). In our study, the age pattern is consistent: the effects are, on average, large, since we hardly observe psychedelic use in middle-aged to old respondents. While some of the age effects are medium in size, the effects of gender on consumption are small to large. The underlying mechanisms for the effects of gender and age might be similar. Prior studies on other forms of substance use, for example on (underage) drinking,^71,72^ cannabis use,^73^ or the nonmedical use of prescription drugs,^74^ have revealed that self-control is an important predictor of such behavior. Other work has indicated that males have, on average, lower capabilities to exercise self-control (including to be more risk-prone) and therefore show more criminal or antisocial behavior^75^. Males with exposure to pharmacological substances have also been found to have higher rates of substance use disorders compared to females with similar levels of substance exposure^76^. Self-control is also related to age, whereby exercising self-control increases with age^77^. Also sensation-seeking, a predictor for drug use, might be related to age^78^. It has further been argued that some measures of self-control explain the correlation between gender, age, and deviant behavior^79^. Moreover, those seeking to improve their lives through psychedelics might either seek to enhance their self-control or use psychedelics as a form of self-control in relation to health behavior change^80^.

While another study did not find a statistically significant association between employment and psychedelics use,^81^ our multivariate analysis indicated that respondents employed part-time had a higher probability to report a higher six-month prevalence rate for all three measures. On the one hand, part-time employment might be associated with more microdosing because the pressure to perform well in fixed-term and not well-paid employment contracts is higher compared to permanent contracts. On the other hand, part-time employment results in more spare time and less opportunity costs for medium to high dose and recreational drug use. A recent U.S. study found being uninsured was related to higher usage rates of psychedelics^69^. As health insurance is typically linked to one’s employer in the U.S., this could perhaps be considered another measure of secure employment. The authors also noted that these data could represent users who are particularly motivated by self-medication, lacking affordable access to the healthcare system.

A high compared to low household income was associated with a lower probability of a six-month prevalence for any dosage, an effect also seen when isolating medium to large doses (here the prevalence was also lower for high compared to medium income), but not with microdoses. While some other studies also did not observe significant differences in the odds of using certain psychedelics,^70^ some similarities to our observed effects were found in recent research, reporting that low income was correlated, for example, with LSD and hallucinogen use^66,67,82^. A possible interpretation is that respondents living in low income households have more stress because of the challenges of relative poverty and accordingly less alternative resources to cope with stress. Another possible interpretation could be that psychedelics are a less expensive solution than conventional therapy, and therefore more attractive to those with less income^33^. Perhaps, therefore, they present a greater demand for microdoses of psychedelics as cognitive enhancers. However, in the sensitivity analysis, the small income effects slightly decreased in size and turned to non-substantial effects, while, interestingly, past six-month microdosing appeared more likely in the highest compared to the lowest income group. Thus, the findings should be interpreted with caution. Future work on reasons for use and willingness to report use could help explore this relationship.

While we found some effects of education in the bivariate analyses, such that higher education levels were associated with increased rates of psychedelic use,^[cf., 66,67,70^ these effects do not remain substantial in the multivariate analysis. It is possible that the strong effect of age, with younger respondents generally more likely to have used, could explain this relationship. However, future work should continue to investigate the relationship between education level and psychedelic use. A U.S. study on psychedelic users reported higher accuracy in knowledge about the substances in those with higher levels of education^83^. This study did not address usage rates, as it focused on psychedelic users, but did uncover some potential effects of education on factors related to use.

Respondents living in a relationship had a lower probability to consume psychedelics in the past six months and for microdosing, in particular,^[cf., 67^ where the effects were large in the multivariate analysis. The effects of partnership on lifetime prevalence were non-substantial. Similar to income, it might be that being in a partnership provides other resources to cope with stress^84,85^. It has further been suggested that a partner enhances socialization and so deters risky and illegal behavior^86,87^. Finally, according to the turning point approach^88^, engaging in a stable partnership or marital cohesion can be described as a crucial turning point in life, changing the opportunity structure and increasing the opportunity costs of deviant and illegal activities in general.

Moreover, living in rural areas of Germany decreased the probability of using psychedelics only for microdosing within the past six months;^[cf., 67^ all other effects of place of residence are non-substantial in the multivariate models. While previous research has suggested a general effect of the urban environment supporting greater access to drugs^89^, our findings suggest a relatively selective increase in use. Perhaps the urban environment allows greater access to the specific venues and groups of individuals who are interested in drug use^90^, in this case, a newer form of psychedelics use.

### Limitations and future research

One limitation of this study is the use of self-report measures since they are prone to social desirability bias. However, the use of web-based surveys may reduce such bias^91^ since they provide more anonymity^92^. Moreover, only very few respondents used the response option “no response” (at most 0.9% for any item), although this selection frequency is similar to, or even greater than other selections (e.g., prevalence of past six-month LSD use). Drop-out on this page was very low (*N* = 10, 0.08%). In addition, sensitivity analyses which control for anonymity perceptions of the survey^56^ led only to minimal changes of the results. These results thus suggest that social desirability bias might not be severe.

While this study differentiates between microdosing and medium to high dosing as two distinct categories, future research may use more nuanced measures with more categories^27,28^. However, until full legalization and therefore accurate and measurable titration of psychedelic dose, it might be difficult for users outside of clinical settings to know which exact dose they actually took or should take. Therefore, future studies may also ask about use frequency^93^ (e.g., daily vs. irregular trips), estimates of specific dosage amounts, intended dose, or types of psychedelic experience (e.g., subperceptual vs. an actual high).

The analysis concerning the socio-demographic variation in the past six-month prevalence needs to be interpreted with caution given the partially very low number of psychedelic users, especially for microdosing. Although our sample size can be considered large, future studies may use even larger samples to replicate and expand upon the findings. Moreover, we present cross-sectional data in one country, while future studies should also use longitudinal designs in different socio-cultural contexts to examine similarities and differences in the pattern found here. Also, the influence of the recent legalization of cannabis in Germany on the use of other substances might be worth investigating^94^.

Given the evidence that younger individuals engage in psychedelic use, it is also important to examine possible socio-demographic associations with such use. Future studies should include youth (under the age of 18), who may show different patterns of use than adults. These findings may have implications for educational interventions tailored towards pediatric and young adult populations.

Intensive studies have identified myriad reasons for use, including recreational, spiritual, therapeutic, and self-enhancement^6.^ To better understand these patterns of desired outcomes as well as the actual vs. desired effects (including whether incorrect microdosing may result in unpleasant full-blown trips),^59^ population-level details regarding use are critical. Questions remain regarding psychedelics’ efficacy for the range of outcomes people anticipate when they use the substances.

## Conclusion

Despite increasing research interest in psychedelics and potential therapeutic applications in controlled settings, in the German context, psychedelic use seems currently relatively low. Still, we encourage further monitoring of potential use trends to understand if factors such as research interest and more therapeutic uses together with increasing media attention also increase the prevalence in the future. Moreover, our analyses uncovered high probabilities of using multiple forms of psychedelics as well as variation in use across socio-demographic groups. In sum, these findings provide foundational information, key to understanding the state and pattern of current use of psychedelics, that should be considered when developing policies and interventions for psychedelic use. The great deal of overlap found in users of psilocybin and LSD suggests that both substances should be given consideration when deciding upon new potential regulations, rather than excluding LSD from the discussion.

## Electronic supplementary material

Below is the link to the electronic supplementary material.

Supplementary Material 1

## Data Availability

The data analyzed during the current study are available at the repository of Bielefeld University^95^.
