# Supplementary material for: Population-based estimates of different dosage types of psychedelic use across socio-demographic groups in Germany
Source: Sci Rep. 2025 May 29;15:18952. doi: 10.1038/s41598-025-03873-0 (PMC12122786; doi:10.1038/s41598-025-03873-0)
Supplement: Supplementary file 1 — Supplementary Material 1 [file 41598_2025_3873_MOESM1_ESM.docx]

Sattler, S., Wood, S., Petersen, M. A., Seiffert, F., Mehlkop, G. (2025) Population-based estimates of different dosage types of psychedelic use across socio-demographic groups in Germany. ***Scientific Reports*** 15. <https://doi.org/10.1038/s41598-025-03873-0>

# Supplementary Material

**Table S1: Unadjusted odds ratios (*UOR*) and adjusted odds ratios (*AOR,* each with 95% confidence intervals in brackets) for any dosing over the lifetime – alternating reference groups^a^ (*N*=11,299)**

|  | ***UORs*** | | | | | | | |
| --- | --- | --- | --- | --- | --- | --- | --- | --- |
| **Age** | Reference: 18-29 | Reference: 30-39 | Reference: 40-49 | | Reference: 50-59 | | Reference: 60+ | |
| 18-29 |  | 0.86 [0.63;1.18] | 1.27 [0.89;1.84] | | ***2.30*** [1.59;3.34] | | **5.39** [3.50;8.29] | |
| 30-39 | 1.16 [0.84;1.59] |  | 1.48 [1.09;2.00] | | ***2.67*** [1.96;3.64] | | **6.24** [4.27;9.13] | |
| 40-49 | 0.78 [0.54;1.13] | 0.68 [0.50;0.92] |  | | *1.81* [1.26;2.59] | | **4.22** [2.77;6.43] | |
| 50-59 | ***0.43*** [0.30;0.63] | ***0.37*** [0.27;0.51] | *0.55* [0.39;0.79] | |  | | ***2.34*** [1.53;3.58] | |
| 60+ | **0.19** [0.12;0.29] | **0.16** [0.11;0.23] | **0.24** [0.16;0.36] | | ***0.43*** [0.28;0.66] | |  | |
| **Employment status** | Reference:  Full-time | Reference:  Part-time | Reference:  In education | | Reference:  Not employed | |  | |
| Full-time |  | 1.24 [0.94;1.64] | 0.90 [0.60;1.36] | | ***2.26*** [1.60;3.19] | |  | |
| Part-time | 0.81 [0.61;1.07] |  | 0.73 [0.46;1.15] | | *1.82* [1.21;2.73] | |  | |
| In education | 1.11 [0.74;1.68] | 1.38 [0.87;2.18] |  | | ***2.51*** [1.51;4.15] | |  | |
| Not employed | ***0.44*** [0.31;0.63] | *0.55* [0.37;0.82] | ***0.40*** [0.24;0.66] | |  | |  | |
| **Equivalence income** | Reference: Low | Reference: Medium | Reference: High | |  | |  | |
| Low (<60% median) |  | 0.99 [0.74;1.32] | 0.77 [0.47;1.25] | |  | |  | |
| Medium | 1.01 [0.76;1.35] |  | 0.78 [0.51;1.19] | |  | |  | |
| High (>2*median) | 1.30 [0.80;2.11] | 1.28 [0.84;1.96] |  | |  | |  | |
|  | ***AORs*** | | | | | | | |
| **Age** | Reference: 18-29 | Reference: 30-39 | | Reference: 40-49 | | Reference: 50-59 | | Reference: 60+ |
| 18-29 |  | 0.84 [0.59;1.22] | | 1.22 [0.78;1.91] | | ***2.22*** [1.43;3.44] | | **5.75** [3.26;10.13] |
| 30-39 | 1.18 [0.82;1.71] |  | | 1.45 [1.05;1.99] | | ***2.63*** [1.90;3.64] | | **6.81** [4.16;11.13] |
| 40-49 | 0.82 [0.52;1.28] | 0.69 [0.50;0.95] | |  | | *1.82* [1.26;2.61] | | **4.71** [2.70;8.22] |
| 50-59 | ***0.45*** [0.29;0.70] | ***0.38*** [0.27;0.53] | | *0.55* [0.38;0.79] | |  | | ***2.59*** [1.54;4.35] |
| 60+ | **0.17** [0.10;0.31] | **0.15** [0.09;0.24] | | **0.21** [0.12;0.37] | | ***0.39*** [0.23;0.65] | |  |
| **Employment status** | Reference:  Full-time | Reference:  Part-time | | Reference:  In education | | Reference:  Not employed | |  |
| Full-time |  | 0.85 [0.63;1.15] | | 1.18 [0.72;1.92] | | 0.82 [0.52;1.28] | |  |
| Part-time | 1.17 [0.87;1.58] |  | | 1.38 [0.78;2.42] | | 0.96 [0.60;1.52] | |  |
| In education | 0.85 [0.52;1.39] | 0.73 [0.41;1.27] | |  | | 0.70 [0.37;1.32] | |  |
| Not employed | 1.22 [0.78;1.92] | 1.04 [0.66;1.65] | | 1.44 [0.76;2.73] | |  | |  |
| **Equivalence income** | Reference: Low | Reference: Medium | | Reference: High | |  | |  |
| Low (<60% median) |  | 0.94 [0.70;1.27] | | 0.88 [0.53;1.46] | |  | |  |
| Medium | 1.06 [0.79;1.43] |  | | 0.93 [0.60;1.45] | |  | |  |
| High (>2*median) | 1.14 [0.69;1.88] | 1.07 [0.69;1.67] | |  | |  | |  |

*Notes:* ^a^Through rotation of the respective reference group, we test for all possible differences between different groups. *UORs* refer to bivariate comparisons between groups, while *AORs* control for the other independent variables. Small effects (*OR*≥1.50 and ≤0.67) are displayed in *italics*, medium effects (*OR*≥2 and ≤0.5) in ***bolded italics***, and large effects (*OR*≥3 and ≤0.33) in **bold**.

**Table S2: Unadjusted odds ratios (*UOR*) and adjusted odds ratios (*AOR,* each with 95% confidence intervals in brackets) for any dosing over the past six months – alternating reference groups^a^ (*N*=11,299)**

|  | ***UORs*** | | | | |
| --- | --- | --- | --- | --- | --- |
| **Age** | Reference: 18-29 | Reference: 30-39 | Reference: 40-49 | Reference: 50-59 | Reference: 60+ |
| 18-29 |  | *1.92* [0.91;4.03] | **5.57** [2.36;13.15] | **20.26** [7.40;55.45] | **63.76** [14.08;288.64] |
| 30-39 | *0.52* [0.25;1.10] |  | ***2.91*** [1.36;6.23] | **10.57** [4.19;26.68] | **33.27** [7.75;142.88] |
| 40-49 | **0.18** [0.08;0.42] | ***0.34*** [0.16;0.74] |  | **3.64** [1.31;10.10] | **11.44** [2.50;52.32] |
| 50-59 | **0.05** [0.02;0.14] | **0.09** [0.04;0.24] | **0.27** [0.10;0.76] |  | **3.15** [0.63;15.71] |
| 60+ | **0.02** [0.00;0.07] | **0.03** [0.01;0.13] | **0.09** [0.02;0.40] | **0.32** [0.06;1.59] |  |
| **Employment status** | Reference:  Full-time | Reference:  Part-time | Reference:  In education | Reference:  Not employed |  |
| Full-time |  | *0.63* [0.26;1.53] | ***0.38*** [0.18;0.82] | **3.60** [1.30;9.97] |  |
| Part-time | *1.60* [0.65;3.89] |  | *0.61* [0.22;1.69] | **5.74** [1.69;19.54] |  |
| In education | ***2.61*** [1.22;5.57] | *1.63* [0.59;4.52] |  | **9.38** [3.02;29.11] |  |
| Not employed | **0.28** [0.10;0.77] | **0.17** [0.05;0.59] | **0.11** [0.03;0.33] |  |  |
| **Equivalence income** | Reference: Low | Reference: Medium | Reference: High |  |  |
| Low (<60% median) |  | ***2.01*** [1.01;3.99] | ***2.40*** [0.65;8.87] |  |  |
| Medium | ***0.50*** [0.25;0.99] |  | 1.19 [0.33;4.32] |  |  |
| High (>2*median) | ***0.42*** [0.11;1.54] | 0.84 [0.23;3.03] |  |  |  |
|  | ***AORs*** | | | | |
| **Age** | Reference: 18-29 | Reference: 30-39 | Reference: 40-49 | Reference: 50-59 | Reference: 60+ |
| 18-29 |  | 1.19 [0.52;2.72] | **3.40** [1.20;9.64] | **12.71** [3.69;43.83] | **40.13** [7.00;230.05] |
| 30-39 | 0.84 [0.37;1.92] |  | ***2.86*** [1.26;6.50] | **10.69** [3.93;29.08] | **33.75** [7.05;161.60] |
| 40-49 | **0.29** [0.10;0.84] | ***0.35*** [0.15;0.80] |  | **3.74** [1.34;10.43] | **11.81** [2.30;60.68] |
| 50-59 | **0.08** [0.02;0.27] | **0.09** [0.03;0.25] | **0.27** [0.10;0.74] |  | **3.16** [0.58;17.13] |
| 60+ | **0.02** [0.00;0.14] | **0.03** [0.01;0.14] | **0.08** [0.02;0.43] | **0.32** [0.06;1.72] |  |
| **Employment status** | Reference:  Full-time | Reference:  Part-time | Reference:  In education | Reference:  Not employed |  |
| Full-time |  | *0.51* [0.21;1.26] | 1.25 [0.51;3.07] | 0.96 [0.31;2.95] |  |
| Part-time | *1.95* [0.79;4.79] |  | ***2.44*** [0.75;7.89] | *1.88* [0.55;6.37] |  |
| In education | 0.80 [0.33;1.96] | ***0.41*** [0.13;1.33] |  | 0.77 [0.19;3.06] |  |
| Not employed | 1.04 [0.34;3.17] | *0.53* [0.16;1.81] | 1.30 [0.33;5.16] |  |  |
| **Equivalence income** | Reference: Low | Reference: Medium | Reference: High |  |  |
| Low (<60% median) |  | 1.28 [0.57;2.89] | *1.77* [0.48;6.60] |  |  |
| Medium | 0.78 [0.35;1.76] |  | 1.38 [0.38;5.04] |  |  |
| High (>2*median) | *0.56* [0.15;2.10] | 0.72 [0.20;2.63] |  |  |  |

*Notes:* ^a^Through rotation of the respective reference group, we test for all possible differences between different groups. *UORs* refer to bivariate comparisons between groups, while *AORs* control for the other independent variables. Small effects (*OR*≥1.50 and ≤0.67) are displayed in *italics*, medium effects (*OR*≥2 and ≤0.5) in ***bolded italics***, and large effects (*OR*≥3 and ≤0.33) in **bold**.

**Table S3: Unadjusted odds ratios (*UOR*) and adjusted odds ratios (*AOR,* each with 95% confidence intervals in brackets) for microdosing over the lifetime – alternating reference groups^a^ (*N*=11,299)**

|  | ***UORs*** | | | | | |
| --- | --- | --- | --- | --- | --- | --- |
| **Age** | Reference: 18-29 | Reference: 30-39 | Reference: 40-49 | Reference: 50-59 | Reference: 60+ |  |
| 18-29 |  | 0.70 [0.43;1.13] | 1.12 [0.68;1.86] | *1.75* [1.03;2.97] | **5.13** [2.79;9.46] |  |
| 30-39 | 1.43 [0.88;2.33] |  | *1.61* [1.12;2.30] | ***2.51*** [1.70;3.70] | **7.36** [4.48;12.08] |  |
| 40-49 | 0.89 [0.54;1.48] | *0.62* [0.44;0.89] |  | *1.56* [1.03;2.37] | **4.58** [2.73;7.68] |  |
| 50-59 | *0.57* [0.34;0.97] | ***0.40*** [0.27;0.59] | *0.64* [0.42;0.97] |  | ***2.93*** [1.71;5.02] |  |
| 60+ | **0.19** [0.11;0.36] | **0.14** [0.08;0.22] | **0.22** [0.13;0.37] | ***0.34*** [0.20;0.58] |  |  |
| **Employment status** | Reference:  Full-time | Reference:  Part-time | Reference:  In education | Reference:  Not employed |  |  |
| Full-time |  | 1.21 [0.80;1.82] | 0.84 [0.49;1.43] | **2.20** [1.43;3.38] |  |  |
| Part-time | 0.83 [0.55;1.25] |  | 0.69 [0.37;1.30] | *1.82* [1.06;3.12] |  |  |
| In education | 1.20 [0.70;2.05] | 1.45 [0.77;2.71] |  | **2.63** [1.38;5.01] |  |  |
| Not employed | ***0.46*** [0.30;0.70] | *0.55* [0.32;0.94] | ***0.38*** [0.20;0.72] |  |  |  |
| **Equivalence income** | Reference: Low | Reference: Medium | Reference: High |  |  |  |
| Low (<60% median) |  | 1.07 [0.75;1.53] | 1.08 [0.58;2.03] |  |  |  |
| Medium | 0.94 [0.65;1.34] |  | 1.01 [0.57;1.79] |  |  |  |
| High (>2*median) | 0.92 [0.49;1.73] | 0.99 [0.56;1.74] |  |  |  |  |
|  | ***AORs*** | | | | | |
| **Age** | Reference: 18-29 | Reference: 30-39 | Reference: 40-49 | Reference: 50-59 | Reference: 60+ |  |
| 18-29 |  | *0.66* [0.39;1.15] | 1.06 [0.58;1.92] | *1.67* [0.92;3.03] | **5.50** [2.51;12.05] |  |
| 30-39 | *1.51* [0.87;2.59] |  | *1.59* [1.08;2.35] | ***2.51*** [1.66;3.79] | **8.27** [4.45;15.37] |  |
| 40-49 | 0.94 [0.52;1.71] | *0.63* [0.43;0.93] |  | *1.57* [1.04;2.37] | **5.19** [2.80;9.61] |  |
| 50-59 | *0.60* [0.33;1.09] | ***0.40*** [0.26;0.60] | *0.64* [0.42;0.96] |  | **3.30** [1.70;6.40] |  |
| 60+ | **0.18** [0.08;0.40] | **0.12** [0.07;0.22] | **0.19** [0.10;0.36] | **0.30** [0.16;0.59] |  |  |
| **Employment status** | Reference:  Full-time | Reference:  Part-time | Reference:  In education | Reference:  Not employed |  |  |
| Full-time |  | 0.85 [0.55;1.32] | 0.88 [0.45;1.74] | 0.80 [0.46;1.37] |  |  |
| Part-time | 1.18 [0.76;1.83] |  | 1.04 [0.44;2.47] | 0.94 [0.53;1.67] |  |  |
| In education | 1.13 [0.57;2.25] | 0.96 [0.40;2.29] |  | 0.91 [0.39;2.12] |  |  |
| Not employed | 1.25 [0.73;2.15] | 1.06 [0.60;1.88] | 1.10 [0.47;2.58] |  |  |  |
| **Equivalence income** | Reference: Low | Reference: Medium | Reference: High |  |  |  |
| Low (<60% median) |  | 1.05 [0.71;1.56] | 1.26 [0.66;2.43] |  |  |  |
| Medium | 0.95 [0.64;1.41] |  | 1.20 [0.68;2.13] |  |  |  |
| High (>2*median) | 0.79 [0.41;1.52] | 0.83 [0.47;1.47] |  |  |  |  |

*Notes:* ^a^Through rotation of the respective reference group, we test for all possible differences between different groups. *UORs* refer to bivariate comparisons between groups, while *AORs* control for the other independent variables. Small effects (*OR*≥1.50 and ≤0.67) are displayed in *italics*, medium effects (*OR*≥2 and ≤0.5) in ***bolded italics***, and large effects (*OR*≥3 and ≤0.33) in **bold**.

**Table S4: Unadjusted odds ratios (*UOR*) and adjusted odds ratios (*AOR,* each with 95% confidence intervals in brackets) for microdosing over the past six months – alternating reference groups^a^ (*N*=11,299)**

|  | ***UORs*** | | | | |
| --- | --- | --- | --- | --- | --- |
| **Age** | Reference: 18-29 | Reference: 30-39 | Reference: 40-49 | Reference: 50-59 | Reference: 60+ |
| 18-29 |  | ***2.00*** [0.53;7.46] | **3.72** [0.99;13.92] | **11.28** [2.80;45.36] | **30.15** [5.23;173.90] |
| 30-39 | ***0.50*** [0.13;1.87] |  | *1.86* [0.63;5.54] | **5.65** [1.74;18.32] | **15.10** [3.09;73.82] |
| 40-49 | **0.27** [0.07;1.01] | *0.54* [0.18;1.60] |  | **3.03** [0.93;9.85] | **8.10** [1.66;39.66] |
| 50-59 | **0.09** [0.02;0.36] | **0.18** [0.05;0.57] | **0.33** [0.10;1.07] |  | ***2.67*** [0.51;13.90] |
| 60+ | **0.03** [0.01;0.19] | **0.07** [0.01;0.32] | **0.12** [0.03;0.60] | ***0.37*** [0.07;1.95] |  |
| **Employment status** | Reference:  Full-time | Reference:  Part-time | Reference:  In education | Reference:  Not employed |  |
| Full-time |  | ***0.38*** [0.11;1.35] | ***0.38*** [0.12;1.17] | **4.10** [1.10;15.38] |  |
| Part-time | ***2.63*** [0.74;9.31] |  | 0.99 [0.24;4.14] | **10.77** [2.22;52.38] |  |
| In education | ***2.64*** [0.85;8.18] | 1.01 [0.24;4.19] |  | **10.84** [2.48;47.39] |  |
| Not employed | **0.24** [0.07;0.91] | **0.09** [0.02;0.45] | **0.09** [0.02;0.40] |  |  |
| **Equivalence income** | Reference: Low | Reference: Medium | Reference: High |  |  |
| Low (<60% median) |  | *1.64* [0.52;5.19] | 1.46 [0.26;8.21] |  |  |
| Medium | *0.61* [0.19;1.94] |  | 0.89 [0.17;4.66] |  |  |
| High (>2*median) | 0.68 [0.12;3.84] | 1.12 [0.21;5.83] |  |  |  |
|  | ***AORs*** | | | | |
| **Age** | Reference: 18-29 | Reference: 30-39 | Reference: 40-49 | Reference: 50-59 | Reference: 60+ |
| 18-29 |  | 0.98 [0.27;3.50] | *1.81* [0.42;7.92] | **5.73** [1.10;29.95] | **14.08** [2.07;95.79] |
| 30-39 | 1.02 [0.29;3.68] |  | *1.86* [0.55;6.26] | **5.87** [1.54;22.35] | **14.43** [2.71;76.83] |
| 40-49 | *0.55* [0.13;2.40] | *0.54* [0.16;1.81] |  | **3.16** [0.97;10.27] | **7.76** [1.49;40.45] |
| 50-59 | **0.17** [0.03;0.91] | **0.17** [0.04;0.65] | **0.32** [0.10;1.03] |  | ***2.46*** [0.45;13.39] |
| 60+ | **0.07** [0.01;0.48] | **0.07** [0.01;0.37] | **0.13** [0.02;0.67] | ***0.41*** [0.07;2.22] |  |
| **Employment status** | Reference:  Full-time | Reference:  Part-time | Reference:  In education | Reference:  Not employed |  |
| Full-time |  | **0.29** [0.08;1.00] | 1.03 [0.26;4.15] | 1.23 [0.26;5.88] |  |
| Part-time | **3.47** [1.00;12.00] |  | **3.58** [0.58;21.94] | **4.26** [1.02;17.77] |  |
| In education | 0.97 [0.24;3.89] | **0.28** [0.05;1.71] |  | 1.19 [0.19;7.46] |  |
| Not employed | 0.81 [0.17;3.90] | **0.23** [0.06;0.98] | 0.84 [0.13;5.27] |  |  |
| **Equivalence income** | Reference: Low | Reference: Medium | Reference: High |  |  |
| Low (<60% median) |  | 0.90 [0.23;3.47] | 0.85 [0.16;4.45] |  |  |
| Medium | 1.11 [0.29;4.29] |  | 0.94 [0.18;4.86] |  |  |
| High (>2*median) | 1.18 [0.22;6.21] | 1.06 [0.21;5.48] |  |  |  |

*Notes:* ^a^Through rotation of the respective reference group, we test for all possible differences between different groups. *UORs* refer to bivariate comparisons between groups, while *AORs* control for the other independent variables. Small effects (*OR*≥1.50 and ≤0.67) are displayed in *italics*, medium effects (*OR*≥2 and ≤0.5) in ***bolded italics***, and large effects (*OR*≥3 and ≤0.33) in **bold**.

**Table S5: Unadjusted odds ratios (*UOR*) and adjusted odds ratios (*AOR,* each with 95% confidence intervals in brackets) for medium to high dosing over the lifetime – alternating reference groups^a^ (*N*=11,299)**

|  | ***UORs*** | | | | | |
| --- | --- | --- | --- | --- | --- | --- |
| **Age** | Reference: 18-29 | Reference: 30-39 | Reference: 40-49 | Reference: 50-59 | Reference: 60+ |  |
| 18-29 |  | 0.87 [0.61;1.23] | 1.29 [0.86;1.94] | ***2.99*** [1.94;4.63] | **6.45** [3.85;10.82] |  |
| 30-39 | 1.15 [0.81;1.64] |  | 1.49 [1.06;2.09] | **3.45** [2.37;5.01] | **7.44** [4.67;11.84] |  |
| 40-49 | 0.78 [0.52;1.17] | *0.67* [0.48;0.95] |  | ***2.32*** [1.51;3.56] | **5.01** [3.01;8.33] |  |
| 50-59 | **0.33** [0.22;0.52] | **0.29** [0.20;0.42] | ***0.43*** [0.28;0.66] |  | ***2.16*** [1.27;3.67] |  |
| 60+ | **0.15** [0.09;0.26] | **0.13** [0.08;0.21] | **0.20** [0.12;0.33] | ***0.46*** [0.27;0.79] |  |  |
| **Employment status** | Reference:  Full-time | Reference:  Part-time | Reference:  In education | Reference:  Not employed |  |  |
| Full-time |  | 1.22 [0.88;1.68] | 1.03 [0.66;1.61] | ***2.29*** [1.53;3.42] |  |  |
| Part-time | 0.82 [0.59;1.14] |  | 0.85 [0.51;1.41] | *1.88* [1.17;3.01] |  |  |
| In education | 0.97 [0.62;1.51] | 1.18 [0.71;1.96] |  | ***2.22*** [1.26;3.88] |  |  |
| Not employed | ***0.44*** [0.29;0.66] | *0.53* [0.33;0.85] | ***0.45*** [0.26;0.79] |  |  |  |
| **Equivalence income** | Reference: Low | Reference: Medium | Reference: High |  |  |  |
| Low (<60% median) |  | 1.04 [0.75;1.45] | 0.72 [0.42;1.22] |  |  |  |
| Medium | 0.96 [0.69;1.33] |  | 0.69 [0.44;1.09] |  |  |  |
| High (>2*median) | 1.39 [0.82;2.36] | 1.45 [0.92;2.30] |  |  |  |  |
|  | ***AORs*** | | | | | |
| **Age** | Reference: 18-29 | Reference: 30-39 | Reference: 40-49 | Reference: 50-59 | Reference: 60+ |  |
| 18-29 |  | 0.86 [0.57;1.28] | 1.26 [0.76;2.07] | ***2.97*** [1.77;4.97] | **7.52** [3.87;14.63] |  |
| 30-39 | 1.17 [0.78;1.74] |  | 1.47 [1.02;2.10] | **3.46** [2.33;5.15] | **8.77** [4.86;15.84] |  |
| 40-49 | 0.80 [0.48;1.31] | 0.68 [0.48;0.98] |  | ***2.36*** [1.53;3.64] | **5.98** [3.07;11.66] |  |
| 50-59 | ***0.34*** [0.20;0.56] | **0.29** [0.19;0.43] | ***0.42*** [0.27;0.65] |  | ***2.53*** [1.35;4.74] |  |
| 60+ | **0.13** [0.07;0.26] | **0.11** [0.06;0.21] | **0.17** [0.09;0.33] | ***0.39*** [0.21;0.74] |  |  |
| **Employment status** | Reference:  Full-time | Reference:  Part-time | Reference:  In education | Reference:  Not employed |  |  |
| Full-time |  | 0.78 [0.56;1.10] | 1.48 [0.88;2.49] | 0.73 [0.44;1.21] |  |  |
| Part-time | 1.28 [0.91;1.80] |  | *1.89* [1.03;3.47] | 0.93 [0.55;1.57] |  |  |
| In education | 0.68 [0.40;1.14] | *0.53* [0.29;0.97] |  | ***0.49*** [0.24;0.99] |  |  |
| Not employed | 1.38 [0.82;2.30] | 1.08 [0.64;1.82] | ***2.03*** [1.01;4.09] |  |  |  |
| **Equivalence income** | Reference: Low | Reference: Medium | Reference: High |  |  |  |
| Low (<60% median) |  | 0.98 [0.70;1.37] | 0.80 [0.46;1.38] |  |  |  |
| Medium | 1.02 [0.73;1.43] |  | 0.82 [0.51;1.31] |  |  |  |
| High (>2*median) | 1.26 [0.73;2.18] | 1.23 [0.76;1.97] |  |  |  |  |

*Notes:* ^a^Through rotation of the respective reference group, we test for all possible differences between different groups. *UORs* refer to bivariate comparisons between groups, while *AORs* control for the other independent variables. Small effects (*OR*≥1.50 and ≤0.67) are displayed in *italics*, medium effects (*OR*≥2 and ≤0.5) in ***bolded italics***, and large effects (*OR*≥3 and ≤0.33) in **bold**.

**Table S6: Unadjusted odds ratios (*UOR*) and adjusted odds ratios (*AOR,* each with 95% confidence intervals in brackets) for medium to high dosing over the past six months – alternating reference groups^a^ (*N*=11,299 or 7,921^b^)**

|  | ***UORs*** | | | | |
| --- | --- | --- | --- | --- | --- |
| **Age** | Reference: 18-29 | Reference: 30-39 | Reference: 40-49 | Reference: 50-59 | Reference: 60+ |
| 18-29 |  | ***2.09*** [0.96;4.53] | **9.04** [3.41;23.98] | **31.01** [9.73;98.82] | **31.01** [9.73;98.82] |
| 30-39 | ***0.48*** [0.22;1.04] |  | **4.33** [1.76;10.63] | **14.85** [4.97;44.39] | **14.85** [4.97;44.39] |
| 40-49 | **0.11** [0.04;0.29] | **0.23** [0.09;0.57] |  | **3.43** [0.99;11.91] | **3.43** [0.99;11.91] |
| 50-59 | **0.03** [0.01;0.10] | **0.07** [0.02;0.20] | **0.29** [0.08;1.01] |  |  |
| 60+ |  |  |  |  |  |
| **Employment status** | Reference:  Full-time | Reference:  Part-time | Reference:  In education | Reference:  Not employed |  |
| Full-time |  | *0.56* [0.22;1.48] | ***0.34*** [0.15;0.78] | **3.87** [1.14;13.12] |  |
| Part-time | *1.77* [0.68;4.64] |  | *0.61* [0.21;1.79] | **6.85** [1.67;28.10] |  |
| In education | ***2.90*** [1.29;6.54] | *1.64* [0.56;4.81] |  | **11.22** [3.02;41.76] |  |
| Not employed | **0.26** [0.08;0.88] | **0.15** [0.04;0.60] | **0.09** [0.02;0.33] |  |  |
| **Equivalence income** | Reference: Low | Reference: Medium | Reference: High |  |  |
| Low (<60% median) |  | ***2.08*** [0.98;4.43] | ***2.82*** [0.60;13.27] |  |  |
| Medium | ***0.48*** [0.23;1.02] |  | 1.35 [0.29;6.28] |  |  |
| High (>2*median) | ***0.35*** [0.08;1.67] | 0.74 [0.16;3.43] |  |  |  |
|  | ***AORs*** | | | | |
| **Age** | Reference: 18-29 | Reference: 30-39 | Reference: 40-49 | Reference: 50-59 | Reference: 60+ |
| 18-29 |  | 1.30 [0.54;3.11] | **5.65** [1.74;18.35] | **20.12** [5.06;80.00] | **20.12** [5.06;80.00] |
| 30-39 | 0.77 [0.32;1.84] |  | **4.34** [1.66;11.39] | **15.47** [4.79;49.98] | **15.47** [4.79;49.98] |
| 40-49 | **0.18** [0.05;0.58] | **0.23** [0.09;0.60] |  | **3.56** [1.02;12.42] | **3.56** [1.02;12.42] |
| 50-59 | **0.05** [0.01;0.20] | **0.06** [0.02;0.21] | **0.28** [0.08;0.98] |  |  |
| 60+ |  |  |  |  |  |
| **Employment status** | Reference:  Full-time | Reference:  Part-time | Reference:  In education | Reference:  Not employed |  |
| Full-time |  | ***0.45*** [0.17;1.19] | 1.19 [0.47;3.02] | 0.85 [0.24;3.00] |  |
| Part-time | ***2.20*** [0.84;5.74] |  | ***2.62*** [0.78;8.80] | *1.86* [0.46;7.48] |  |
| In education | 0.84 [0.33;2.13] | ***0.38*** [0.11;1.28] |  | 0.71 [0.16;3.17] |  |
| Not employed | 1.18 [0.33;4.19] | *0.54* [0.13;2.16] | 1.41 [0.32;6.29] |  |  |
| **Equivalence income** | Reference: Low | Reference: Medium | Reference: High |  |  |
| Low (<60% median) |  | 1.24 [0.51;3.02] | *1.91* [0.40;9.03] |  |  |
| Medium | 0.80 [0.33;1.95] |  | *1.54* [0.33;7.23] |  |  |
| High (>2*median) | *0.52* [0.11;2.47] | *0.65* [0.14;3.06] |  |  |  |

*Notes:* ^a^Through rotation of the respective reference group, we test for all possible differences between different groups. *UORs* refer to bivariate comparisons between groups, while *AORs* control for the other independent variables. ^b^All *UORs* for the different age categories and all *AORs* are based on 7,921 observations because among respondents aged 60 or older, no one indicated the respective psychedelic drug use. Small effects (*OR*≥1.50 and ≤0.67) are displayed in *italics*, medium effects (*OR*≥2 and ≤0.5) in ***bolded italics***, and large effects (*OR*≥3 and ≤0.33) in **bold**.

**Table S7: Adjusted odds ratios (*AOR*, with 95% confidence intervals in brackets) for psychedelic use over the lifetime and the past six months – controlling for anonymity perceptions^a^ (*N*=11,259^b^)**

|  | **Any dosing** | |  | **Microdosing** | |  | **Medium to high dosing** | |
| --- | --- | --- | --- | --- | --- | --- | --- | --- |
|  | **Lifetime**  *AOR* | **Past six months** *AOR* |  | **Lifetime**  *AOR* | **Past six months** *AOR* |  | **Lifetime**  *AOR* | **Past six months** *AOR* |
| **Sex** |  |  |  |  |  |  |  |  |
| Male (ref.) |  |  |  |  |  |  |  |  |
| Female | *0.58* [0.45,0.73] | ***0.41*** [0.20,0.83] |  | *0.63* [0.46,0.88] | *0.58* [0.19,1.71] |  | ***0.47*** [0.36,0.62] | **0.33** [0.15,0.76] |
| **Age** |  |  |  |  |  |  |  |  |
| 18-29 (ref.) |  |  |  |  |  |  |  |  |
| 30-39 | 1.16 [0.80,1.66] | 0.81 [0.36,1.84] |  | 1.47 [0.85,2.52] | 1.03 [0.30,3.61] |  | 1.14 [0.76,1.69] | 0.74 [0.31,1.73] |
| 40-49 | 0.82 [0.53,1.28] | **0.32** [0.12,0.85] |  | 0.95 [0.53,1.70] | *0.62* [0.16,2.43] |  | 0.80 [0.49,1.31] | **0.19** [0.06,0.59] |
| 50-59 | ***0.45*** [0.29,0.70] | **0.09** [0.03,0.29] |  | *0.60* [0.33,1.08] | **0.21** [0.05,1.01] |  | ***0.34*** [0.20,0.57] | **0.06** [0.01,0.21] |
| 60+ | **0.17** [0.10,0.31] | **0.03** [0.00,0.16] |  | **0.18** [0.08,0.40] | **0.09** [0.01,0.56] |  | **0.13** [0.07,0.26] |  |
| **Education** |  |  |  |  |  |  |  |  |
| Lower than university entrance qualification (ref.) |  |  |  |  |  |  |  |  |
| At least university entrance qualification | 1.10 [0.87,1.40] | 1.07 [0.51,2.26] |  | 1.08 [0.79,1.47] | 1.02 [0.33,3.18] |  | 1.10 [0.84,1.45] | 1.07 [0.46,2.45] |
| **Employment status** |  |  |  |  |  |  |  |  |
| Full-time (ref.) |  |  |  |  |  |  |  |  |
| Part-time | 1.18 [0.88,1.60] | ***2.17*** [0.87,5.41] |  | 1.19 [0.76,1.85] | **4.39** [1.12,17.15] |  | 1.30 [0.92,1.82] | ***2.55*** [0.96,6.75] |
| In education | 0.87 [0.53,1.41] | 0.97 [0.41,2.32] |  | 1.16 [0.59,2.27] | 1.37 [0.36,5.20] |  | 0.70 [0.42,1.17] | 1.07 [0.44,2.62] |
| Not employed | 1.25 [0.80,1.97] | 1.19 [0.38,3.75] |  | 1.27 [0.74,2.20] | 0.98 [0.20,4.75] |  | 1.42 [0.85,2.38] | 1.38 [0.37,5.19] |
| **Equivalence income** |  |  |  |  |  |  |  |  |
| Low (<60% median) (ref.) |  |  |  |  |  |  |  |  |
| Medium | 1.08 [0.80,1.46] | 0.86 [0.37,1.98] |  | 0.97 [0.65,1.44] | 1.27 [0.32,5.04] |  | 1.05 [0.75,1.48] | 0.91 [0.36,2.25] |
| High (>2*median) | 1.17 [0.70,1.94] | 0.69 [0.18,2.64] |  | 0.81 [0.42,1.57] | *1.59* [0.28,9.02] |  | 1.31 [0.75,2.28] | 0.68 [0.14,3.32] |
| **Partner in household** |  |  |  |  |  |  |  |  |
| No partner (ref.) |  |  |  |  |  |  |  |  |
| Partner | 0.93 [0.72,1.19] | ***0.39*** [0.20,0.75] |  | 1.09 [0.80,1.48] | **0.21** [0.08,0.52] |  | 0.86 [0.65,1.14] | ***0.41*** [0.19,0.89] |
| **Place of residence** |  |  |  |  |  |  |  |  |
| Urban (ref.) |  |  |  |  |  |  |  |  |
| Rural | 0.80 [0.62,1.02] | 0.74 [0.34,1.59] |  | 0.93 [0.67,1.27] | *0.60* [0.17,2.10] |  | 0.84 [0.63,1.12] | 0.89 [0.41,1.95] |
| **Anonymity perceptions** | ***0.50*** [0.28,0.89] | **0.07** [0.02,0.27] |  | ***0.50*** [0.23,1.08] | **0.03** [0.01,0.18] |  | ***0.39*** [0.21,0.74] | **0.05** [0.01,0.19] |

*Notes:* ^a^*AORs* control for the other independent variables displayed in the table. ^b^*N*<11,299 due to missing values in the anonymity perceptions variable. The *AORs* for medium to high dosing in the past six months are based on 7,890 observations because among respondents aged 60 or older, no one indicated the respective psychedelic drug use. Small effects (*OR*≥1.50 and ≤0.67) are displayed in *italics*, medium effects (*OR*≥2 and ≤0.5) in ***bolded italics***, and large effects (*OR*≥3 and ≤0.33) in **bold**.

**Table S8: Adjusted odds ratios (*AOR*, with 95% confidence intervals in brackets) for any dosing over the lifetime and past six months – alternating reference groups and controlling for anonymity perceptions^a^ (*N*=11,259^b^)**

|  | ***Lifetime*** | | | | |
| --- | --- | --- | --- | --- | --- |
| **Age** | Reference: 18-29 | Reference: 30-39 | Reference: 40-49 | Reference: 50-59 | Reference: 60+ |
| 18-29 |  | 0.87 [0.60;1.24] | 1.22 [0.78;1.89] | ***2.21*** [1.43;3.41] | **5.75** [3.26;10.12] |
| 30-39 | 1.16 [0.80;1.66] |  | 1.41 [1.03;1.93] | ***2.55*** [1.85;3.53] | **6.64** [4.06;10.85] |
| 40-49 | 0.82 [0.53;1.28] | 0.71 [0.52;0.97] |  | *1.82* [1.26;2.62] | **4.72** [2.70;8.26] |
| 50-59 | ***0.45*** [0.29;0.70] | ***0.39*** [0.28;0.54] | *0.55* [0.38;0.79] |  | ***2.60*** [1.55;4.37] |
| 60+ | **0.17** [0.10;0.31] | **0.15** [0.09;0.25] | **0.21** [0.12;0.37] | ***0.38*** [0.23;0.65] |  |
| **Employment status** | Reference:  Full-time | Reference:  Part-time | Reference:  In education | Reference:  Not employed |  |
| Full-time |  | 0.84 [0.63;1.14] | 1.15 [0.71;1.88] | 0.80 [0.51;1.25] |  |
| Part-time | 1.18 [0.88;1.60] |  | 1.36 [0.78;2.38] | 0.94 [0.60;1.50] |  |
| In education | 0.87 [0.53;1.41] | 0.73 [0.42;1.28] |  | 0.69 [0.36;1.31] |  |
| Not employed | 1.25 [0.80;1.97] | 1.06 [0.67;1.68] | 1.44 [0.76;2.74] |  |  |
| **Equivalence income** | Reference: Low | Reference: Medium | Reference: High |  |  |
| Low (<60% median) |  | 0.93 [0.69;1.26] | 0.86 [0.51;1.42] |  |  |
| Medium | 1.08 [0.80;1.46] |  | 0.92 [0.59;1.43] |  |  |
| High (>2*median) | 1.17 [0.70;1.94] | 1.09 [0.70;1.68] |  |  |  |
|  | ***Past six months*** | | | | |
| **Age** | Reference: 18-29 | Reference: 30-39 | Reference: 40-49 | Reference: 50-59 | Reference: 60+ |
| 18-29 |  | 1.23 [0.54;2.78] | **3.16** [1.18;8.44] | **11.50** [3.49;37.95] | **36.24** [6.28;209.23] |
| 30-39 | 0.81 [0.36;1.84] |  | ***2.57*** [1.14;5.76] | **9.34** [3.49;25.00] | **29.44** [6.12;141.73] |
| 40-49 | **0.32** [0.12;0.85] | ***0.39*** [0.17;0.87] |  | **3.64** [1.29;10.29] | **11.47** [2.20;59.72] |
| 50-59 | **0.09** [0.03;0.29] | **0.11** [0.04;0.29] | **0.27** [0.10;0.78] |  | **3.15** [0.58;17.07] |
| 60+ | **0.03** [0.00;0.16] | **0.03** [0.01;0.16] | **0.09** [0.02;0.45] | **0.32** [0.06;1.72] |  |
| **Employment status** | Reference:  Full-time | Reference:  Part-time | Reference:  In education | Reference:  Not employed |  |
| Full-time |  | ***0.46*** [0.18;1.14] | 1.03 [0.43;2.45] | 0.84 [0.27;2.66] |  |
| Part-time | ***2.17*** [0.87;5.41] |  | ***2.24*** [0.73;6.85] | *1.83* [0.51;6.54] |  |
| In education | 0.97 [0.41;2.32] | ***0.45*** [0.15;1.37] |  | 0.82 [0.19;3.51] |  |
| Not employed | 1.19 [0.38;3.75] | *0.55* [0.15;1.94] | 1.22 [0.28;5.22] |  |  |
| **Equivalence income** | Reference: Low | Reference: Medium | Reference: High |  |  |
| Low (<60% median) |  | 1.16 [0.51;2.68] | 1.46 [0.38;5.59] |  |  |
| Medium | 0.86 [0.37;1.98] |  | 1.25 [0.35;4.44] |  |  |
| High (>2*median) | 0.69 [0.18;2.64] | 0.80 [0.23;2.84] |  |  |  |

*Notes:* ^a^ Through rotation of the respective reference group, we test for all possible differences between different groups. *AORs* control for the other independent variables. ^b^ *N*<11,299 due to missing values in the anonymity perceptions variable. Small effects (*OR*≥1.50 and ≤0.67) are displayed in *italics*, medium effects (*OR*≥2 and ≤0.5) in ***bolded italics***, and large effects (*OR*≥3 and ≤0.33) in **bold**.

**Table S9: Adjusted odds ratios (*AOR*, with 95% confidence intervals in brackets) for microdosing over the lifetime and past six months – alternating reference groups controlling for anonymity perceptions^a^ (*N*=11,259^b^)**

|  | ***Lifetime*** | | | | |
| --- | --- | --- | --- | --- | --- |
| **Age** | Reference: 18-29 | Reference: 30-39 | Reference: 40-49 | Reference: 50-59 | Reference: 60+ |
| 18-29 |  | 0.68 [0.40;1.18] | 1.06 [0.59;1.90] | *1.66* [0.92;2.99] | **5.48** [2.52;11.93] |
| 30-39 | 1.47 [0.85;2.52] |  | *1.55* [1.05;2.28] | ***2.44*** [1.62;3.67] | **8.03** [4.35;14.84] |
| 40-49 | 0.95 [0.53;1.70] | *0.65* [0.44;0.95] |  | *1.57* [1.04;2.37] | **5.18** [2.80;9.58] |
| 50-59 | *0.60* [0.33;1.08] | ***0.41*** [0.27;0.62] | *0.64* [0.42;0.96] |  | **3.30** [1.70;6.39] |
| 60+ | **0.18** [0.08;0.40] | **0.12** [0.07;0.23] | **0.19** [0.10;0.36] | **0.30** [0.16;0.59] |  |
| **Employment status** | Reference:  Full-time | Reference:  Part-time | Reference:  In education | Reference:  Not employed |  |
| Full-time |  | 0.84 [0.54;1.31] | 0.86 [0.44;1.69] | 0.78 [0.45;1.35] |  |
| Part-time | 1.19 [0.76;1.85] |  | 1.03 [0.44;2.40] | 0.93 [0.53;1.65] |  |
| In education | 1.16 [0.59;2.27] | 0.97 [0.42;2.28] |  | 0.91 [0.39;2.12] |  |
| Not employed | 1.27 [0.74;2.20] | 1.07 [0.61;1.90] | 1.10 [0.47;2.57] |  |  |
| **Equivalence income** | Reference: Low | Reference: Medium | Reference: High |  |  |
| Low (<60% median) |  | 1.04 [0.70;1.54] | 1.23 [0.64;2.38] |  |  |
| Medium | 0.97 [0.65;1.44] |  | 1.19 [0.67;2.10] |  |  |
| High (>2*median) | 0.81 [0.42;1.57] | 0.84 [0.48;1.49] |  |  |  |
|  | ***Past six months*** | | | | |
| **Age** | Reference: 18-29 | Reference: 30-39 | Reference: 40-49 | Reference: 50-59 | Reference: 60+ |
| 18-29 |  | 0.97 [0.28;3.37] | *1.61* [0.41;6.33] | **4.66** [0.99;21.86] | **11.69** [1.79;76.19] |
| 30-39 | 1.03 [0.30;3.61] |  | *1.67* [0.51;5.49] | **4.82** [1.33;17.51] | **12.10** [2.35;62.18] |
| 40-49 | *0.62* [0.16;2.43] | *0.60* [0.18;1.97] |  | ***2.89*** [0.86;9.65] | **7.25** [1.39;37.76] |
| 50-59 | **0.21** [0.05;1.01] | **0.21** [0.06;0.75] | ***0.35*** [0.10;1.16] |  | ***2.51*** [0.46;13.71] |
| 60+ | **0.09** [0.01;0.56] | **0.08** [0.02;0.42] | **0.14** [0.03;0.72] | ***0.40*** [0.07;2.18] |  |
| **Employment status** | Reference:  Full-time | Reference:  Part-time | Reference:  In education | Reference:  Not employed |  |
| Full-time |  | **0.23** [0.06;0.89] | 0.73 [0.19;2.76] | 1.02 [0.21;4.97] |  |
| Part-time | **4.39** [1.12;17.15] |  | **3.20** [0.58;17.57] | **4.49** [1.04;19.35] |  |
| In education | 1.37 [0.36;5.20] | **0.31** [0.06;1.72] |  | 1.40 [0.22;9.12] |  |
| Not employed | 0.98 [0.20;4.75] | **0.22** [0.05;0.96] | 0.71 [0.11;4.63] |  |  |
| **Equivalence income** | Reference: Low | Reference: Medium | Reference: High |  |  |
| Low (<60% median) |  | 0.79 [0.20;3.13] | *0.63* [0.11;3.57] |  |  |
| Medium | 1.27 [0.32;5.04] |  | 0.80 [0.16;4.03] |  |  |
| High (>2*median) | *1.59* [0.28;9.02] | 1.25 [0.25;6.32] |  |  |  |

*Notes:* ^a^Through rotation of the respective reference group, we test for all possible differences between different groups. *AORs* control for the other independent variables. ^b^*N*<11,299 due to missing values in the anonymity perceptions variable. Small effects (*OR*≥1.50 and ≤0.67) are displayed in *italics*, medium effects (*OR*≥2 and ≤0.5) in ***bolded italics***, and large effects (*OR*≥3 and ≤0.33) in **bold**.

**Table S10: Adjusted odds ratios (*AOR*, with 95% confidence intervals in brackets) for medium to high dosing over the lifetime and past six months – alternating reference groups controlling for anonymity perceptions^a^ (*N*=11,259^b,c^)**

|  | ***Lifetime*** | | | | | |
| --- | --- | --- | --- | --- | --- | --- |
| **Age** | Reference: 18-29 | Reference: 30-39 | Reference: 40-49 | Reference: 50-59 | | Reference: 60+ |
| 18-29 |  | 0.88 [0.59;1.31] | 1.25 [0.76;2.04] | ***2.94*** [1.77;4.89] | | **7.46** [3.84;14.52] |
| 30-39 | 1.14 [0.76;1.69] |  | 1.42 [0.99;2.03] | **3.34** [2.25;4.95] | | **8.48** [4.70;15.31] |
| 40-49 | 0.80 [0.49;1.31] | 0.71 [0.49;1.01] |  | ***2.36*** [1.52;3.65] | | **5.98** [3.06;11.71] |
| 50-59 | ***0.34*** [0.20;0.57] | **0.30** [0.20;0.44] | ***0.42*** [0.27;0.66] |  | | ***2.54*** [1.36;4.75] |
| 60+ | **0.13** [0.07;0.26] | **0.12** [0.07;0.21] | **0.17** [0.09;0.33] | ***0.39*** [0.21;0.74] | |  |
| **Employment status** | Reference:  Full-time | Reference:  Part-time | Reference:  In education | Reference:  Not employed | |  |
| Full-time |  | 0.77 [0.55;1.08] | 1.43 [0.85;2.40] | 0.70 [0.42;1.18] | |  |
| Part-time | 1.30 [0.92;1.82] |  | *1.86* [1.02;3.38] | 0.91 [0.54;1.55] | |  |
| In education | 0.70 [0.42;1.17] | *0.54* [0.30;0.98] |  | ***0.49*** [0.24;1.00] | |  |
| Not employed | 1.42 [0.85;2.38] | 1.09 [0.65;1.86] | ***2.03*** [1.00;4.11] |  | |  |
| **Equivalence income** | Reference: Low | Reference: Medium | Reference: High |  | |  |
| Low (<60% median) |  | 0.95 [0.68;1.34] | 0.76 [0.44;1.33] |  | |  |
| Medium | 1.05 [0.75;1.48] |  | 0.80 [0.50;1.29] |  | |  |
| High (>2*median) | 1.31 [0.75;2.28] | 1.24 [0.77;2.00] |  |  | |  |
|  | ***Past six months*** | | | | | |
| **Age** | Reference: 18-29 | Reference: 30-39 | Reference: 40-49 | Reference: 50-59 | Reference: 60+ | |
| 18-29 |  | 1.36 [0.58;3.21] | **5.20** [1.70;15.88] | **17.93** [4.67;68.86] | **17.93** [4.67;68.86] | |
| 30-39 | 0.74 [0.31;1.73] |  | **3.82** [1.47;9.92] | **13.18** [4.13;42.06] | **13.18** [4.13;42.06] | |
| 40-49 | **0.19** [0.06;0.59] | **0.26** [0.10;0.68] |  | **3.45** [0.97;12.21] | **3.45** [0.97;12.21] | |
| 50-59 | **0.06** [0.01;0.21] | **0.08** [0.02;0.24] | **0.29** [0.08;1.03] |  |  | |
| 60+ |  |  |  |  |  | |
| **Employment status** | Reference:  Full-time | Reference:  Part-time | Reference:  In education | Reference:  Not employed |  | |
| Full-time |  | ***0.39*** [0.15;1.04] | 0.93 [0.38;2.27] | 0.72 [0.19;2.72] |  | |
| Part-time | ***2.55*** [0.96;6.75] |  | ***2.37*** [0.75;7.54] | *1.84* [0.42;8.09] |  | |
| In education | 1.07 [0.44;2.62] | ***0.42*** [0.13;1.34] |  | 0.78 [0.15;3.92] |  | |
| Not employed | 1.38 [0.37;5.19] | *0.54* [0.12;2.38] | 1.29 [0.25;6.52] |  |  | |
| **Equivalence income** | Reference: Low | Reference: Medium | Reference: High |  |  | |
| Low (<60% median) |  | 1.10 [0.44;2.74] | 1.48 [0.30;7.22] |  |  | |
| Medium | 0.91 [0.36;2.25] |  | 1.34 [0.29;6.12] |  |  | |
| High (>2*median) | 0.68 [0.14;3.32] | 0.75 [0.16;3.43] |  |  |  | |

*Notes:* ^a^Through rotation of the respective reference group, we test for all possible differences between different groups. *AORs* control for the other independent variables. ^b^*N*<11,299 due to missing values in the anonymity perceptions variable. ^c^All adjusted *ORs* for past six months are based on 7,890 observations because among respondents aged 60 or older, no one indicated the respective psychedelic drug use. Small effects (*OR*≥1.50 and ≤0.67) are displayed in *italics*, medium effects (*OR*≥2 and ≤0.5) in ***bolded italics***, and large effects (*OR*≥3 and ≤0.33) in **bold**.

**Table S11: Summary of sizes and directions of effects of logistic regression (adjusted odds ratios)**

|  |  | ***Any dosing*** | ***Microdosing*** | ***Medium to high dosing*** |
| --- | --- | --- | --- | --- |
| **Sex** (Reference: Male) | | | | |
| Lifetime | Female | 🡣 | 🡣 | 🡳 |
| Past six months | Female | 🡳 | 🡣 | 🢃 |
| **Age** (Reference: 18-29) | | | | |
| Lifetime | 30-39 |  | 🡡 (▪) |  |
|  | 40-49 |  |  |  |
|  | 50-59 | 🡳 | 🡣 | 🡳 |
|  | 60+ | 🢃 | 🢃 | 🢃 |
| Past six months | 30-39 |  |  |  |
|  | 40-49 | 🢃 | 🡣 | 🢃 |
|  | 50-59 | 🢃 | 🢃 | 🢃 |
|  | 60+ | 🢃 | 🢃 | N/A |
| **Education** (Reference: Lower than university entrance qualification) | | | | |
| Lifetime | At least university entrance qualification | | | |
| Past six months | At least university entrance qualification | | | |
| **Employment status** (Reference: Full-time) | | | | |
| Lifetime | Part-time |  |  |  |
|  | In education |  |  |  |
|  | Not employed |  |  |  |
| Past six months | Part-time | 🡡 (🡱) | 🢁 | 🡱 |
|  | In education |  |  |  |
|  | Not employed |  |  |  |
| **Equivalence income** (Reference: Low (< 60% median)) | | | | |
| Lifetime | Medium |  |  |  |
|  | High (>2*median) |  |  |  |
| Past six months | Medium |  |  |  |
|  | High (>2*median) | 🡣 (▪) | (🡡) | 🡣 (▪) |
| **Partner in household** (Reference: No partner) | | | | |
| Lifetime | Partner |  |  |  |
| Past six months | Partner | 🡳 | 🢃 | 🡳 |
| **Place of residence** (Reference: Urban) | | | | |
| Lifetime | Rural |  |  |  |
| Past six months | Rural |  | 🡣 |  |

*Notes*: Small red arrows pointing downwards (🡣) indicate a small negative effect, while small green arrows pointing upwards indicate a small positive effect (🡡); mid-sized red arrows pointing downwards (🡳) indicate a medium negative effect; mid-sized green arrows pointing upwards (🡱) indicate a medium positive effect; large red arrows pointing downwards (🢃) indicate a large negative effect; large green arrows pointing upwards (🢁) indicate a large positive effect. Symbols in parentheses indicate the effect size found after considering anonymity perceptions with a sensitivity analysis, if different from the results of the multivariate model, thereby (▪) indicates a non-substantial effect after controlling for anonymity perceptions. N/A=Among respondents aged 60 or older, no one indicated the respective psychedelic drug use.
